# Sustainability in Health care by Allocating Resources Effectively (SHARE) 10: operationalising disinvestment in a conceptual framework for resource allocation

**DOI:** 10.1186/s12913-017-2506-7

**Published:** 2017-09-08

**Authors:** Claire Harris, Sally Green, Adam G. Elshaug

**Affiliations:** 1 School of Public Health and Preventive Medicine, Monash University, Melbourne, Victoria Australia; 2 Centre for Clinical Effectiveness, Monash Health, Melbourne, Victoria Australia; 3 Menzies Centre for Health Policy, Sydney School of Public Health, University of Sydney, Sydney, Australia; 4 Lown Institute, Brookline, Massachusetts USA

**Keywords:** Disinvestment, Decommissioning, De-adoption, Resource allocation, Reinvestment, Reallocation, Rationing, Prioritisation, Decision-making, Framework

## Abstract

**Background:**

This is the tenth in a series of papers reporting a program of Sustainability in Health care by Allocating Resources Effectively (SHARE) in a local healthcare setting. After more than a decade of research, there is little published evidence of active and successful disinvestment. The paucity of frameworks, methods and tools is reported to be a factor in the lack of success. However there are clear and consistent messages in the literature that can be used to inform development of a framework for operationalising disinvestment. This paper, along with the conceptual review of disinvestment in Paper 9 of this series, aims to integrate the findings of the SHARE Program with the existing disinvestment literature to address the lack of information regarding systematic organisation-wide approaches to disinvestment at the local health service level.

**Discussion:**

A framework for disinvestment in a local healthcare setting is proposed. Definitions for essential terms and key concepts underpinning the framework have been made explicit to address the lack of consistent terminology. Given the negative connotations of the word ‘disinvestment’ and the problems inherent in considering disinvestment in isolation, the basis for the proposed framework is ‘resource allocation’ to address the spectrum of decision-making from investment to disinvestment. The focus is positive: optimising healthcare, improving health outcomes, using resources effectively.

The framework is based on three components: a program for decision-making, projects to implement decisions and evaluate outcomes, and research to understand and improve the program and project activities. The program consists of principles for decision-making and settings that provide opportunities to introduce systematic prompts and triggers to initiate disinvestment. The projects follow the steps in the disinvestment process. Potential methods and tools are presented, however the framework does not stipulate project design or conduct; allowing application of any theories, methods or tools at each step. Barriers are discussed and examples illustrating constituent elements are provided.

**Conclusions:**

The framework can be employed at network, institutional, departmental, ward or committee level. It is proposed as an organisation-wide application, embedded within existing systems and processes, which can be responsive to needs and priorities at the level of implementation. It can be used in policy, management or clinical contexts.

**Electronic supplementary material:**

The online version of this article (doi:10.1186/s12913-017-2506-7) contains supplementary material, which is available to authorized users.

## About share

*This is the tenth in a series of papers reporting Sustainability in Health care by Allocating Resources Effectively (SHARE). The SHARE Program is an investigation of concepts, opportunities, methods and implications for evidence-based investment and disinvestment in health technologies and clinical practices in a local healthcare setting. The papers in this series are targeted at clinicians, managers, policy makers, health service researchers and implementation scientists working in this context. This paper proposes a framework for operationalising disinvestment in the context of resource allocation in the local healthcare setting.*

## Background

Although there is no clear single definition, disinvestment is generally understood to be removal, reduction or restriction of technologies and clinical practices (TCPs) that are unsafe or of little benefit, in order to improve patient outcomes and use available resources more efficiently [1]. Three main areas of opportunity for disinvestment have been identified: 1) TCPs in current use that were not evaluated rigorously prior to their introduction and have subsequently been identified as harmful, ineffective or not cost-effective for all patients or certain subgroups, 2) existing TCPs that are safe, effective and cost-effective but which have alternatives offering greater benefit, and 3) TCPs that are overused or misused [1].

Following successful implementation of a systematic, integrated, transparent, evidence-based program to assess new TCPs prior to their introduction within the health service [2], Monash Health, a large health service network in Melbourne Australia, sought to develop a similar program for disinvestment. The ‘Sustainability in Health care by Allocating Resources Effectively’ (SHARE) Program was established to investigate this. An overview of the program and a guide to the SHARE publications are provided in the first paper in this series [3] and a summary of the findings are in the final paper [4].

It is common for healthcare networks and individual facilities to make decisions within organisation-wide frameworks; for example introduction of new TCPs and models of care, delivery of programs and services, development and authorisation of policies and procedures, capital expenditure and clinical purchasing. Although disinvestment can be considered in all these contexts, it is frequently reported in individual standalone projects, isolated from other decision-making settings. Monash Health chose to explore disinvestment in the context of organisation-wide systems and processes for all resource allocation decisions.

**Fig. 1:**
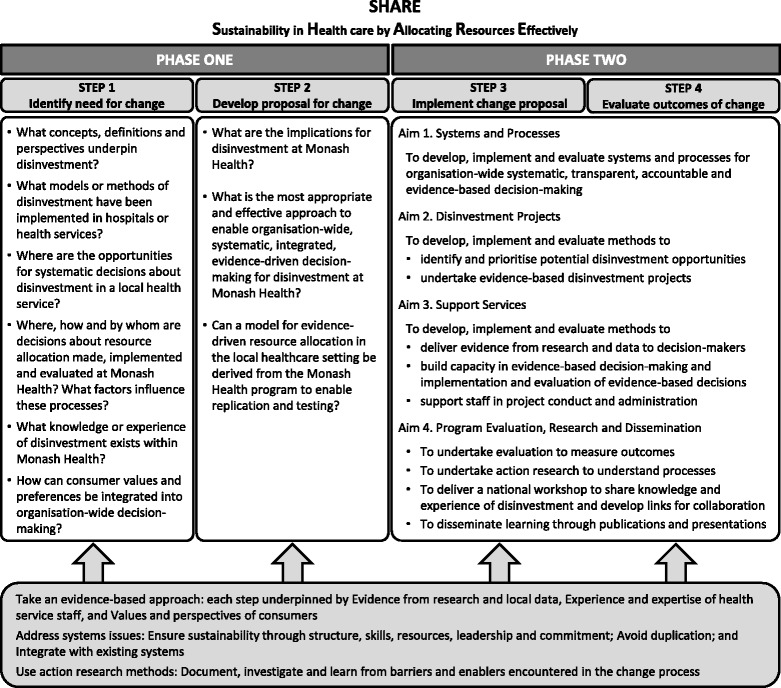
Overview of SHARE Program

There was little published information available to guide development of a systematic organisation-wide local approach to disinvestment at Monash Health. In the absence of guidance from the literature, a two-phased process was proposed to identify and then evaluate potential opportunities for disinvestment (Fig. 1). The aim of Phase One was to understand concepts and practices related to disinvestment and the implications for a local health service and, based on this information, to identify potential settings and methods for decision-making. The aim of Phase Two was to develop, implement and evaluate the proposed settings and methods to determine which were sustainable, effective and appropriate at Monash Health.

The outcomes of Phase One provide information regarding decision-making settings, decision-makers, scope and type of decisions, strengths and weaknesses in current processes, barriers and enablers, and criteria used for allocating resources within a local health service which, to our knowledge, has not previously been documented to this level of detail in this context [5–8]. While the program had limited success in achieving the aims of Phase Two, the investigation provides in-depth insight into the experience of disinvestment in one local health service and reports the process of disinvestment from identification, through prioritisation and decision-making, to implementation and evaluation, and finally explication of the processes and outcomes [9–11]. These detailed findings enabled development of several frameworks and models for a range of purposes related to disinvestment and resource allocation in the local healthcare setting.

At the completion of these activities, a third phase was undertaken to review the current literature from the perspective of a local health service, and combine it with the published findings from the SHARE Program to address some of the gaps in information about disinvestment in this setting. This review focuses on the practical and operational aspects of disinvestment at the local level. It is a companion to the ninth paper of the SHARE series which provides a conceptual description; disinvestment is introduced and discussed in relation to terminology and concepts, motivation and purpose, relationships with other health improvement paradigms, challenges, and implications for policy, practice and research [1]. The methods of the literature review are included in Paper 9 and the contents of both reviews are summarised in Table 1.

**Table 1:** Contents of the literature reviews

SHARE Paper 9. Conceptual perspective
▪ Terminology and concepts – Health technologies – Disinvestment – Resource allocation – Optimising health care – Reinvestment ▪ Motivation and purpose – Impetus for disinvestment – Rationale for disinvestment ▪ Relationships with other healthcare improvement paradigms – Evidence based health care – Quality improvement – System redesign – Health economic approaches ▪ Challenges ▪ New approach to disinvestment
SHARE Paper 10. Operational perspective
▪ Existing theories, frameworks and models ▪ New framework ▪ Program – Principles of decision-making – Settings and opportunities – Prompts and triggers – Steps in the disinvestment process ▪ Projects ▪ Research ▪ Methods and tools – Identification of opportunities – Prioritisation and Decision-making – Development of a proposal – Implementation – Monitoring, Evaluation and Reporting – Reinvestment – Dissemination and Diffusion – Maintenance ▪ Barriers and enablers

Although research and debate has broadened considerably over the past decade, there remains a lack of information to guide healthcare networks or individual facilities in how they might take a systematic, integrated, organisation-wide approach to disinvestment in the context of all resource allocation decisions [1]. Despite the paucity of evidence in this context, there are clear and consistent messages regarding principles for decision-making, settings and opportunities to identify disinvestment targets, steps in the disinvestment process, barriers and enablers to successful implementation, and some frameworks and models for elements of the disinvestment process. This practical information can be used to develop an organisation-wide framework for operationalising disinvestment in the local healthcare setting.

## Aims

The aims of this paper are to discuss the current literature on disinvestment from an operational perspective, combine it with the experiences of the SHARE Program, and propose a framework for disinvestment in the context of resource allocation in the local healthcare setting.

## Existing theories, frameworks and models

### Theories

Theories are based on concepts or ideas that characterise a particular phenomenon and propositions or relationships that link the concepts [12]. No specific theories of disinvestment have been proposed, however resource allocation theory, prioritisation theories, and decision-making theories have been applied in disinvestment projects; examples are listed in Table 2 [13–18].

**Table 2:** Examples of theories proposed or applied in disinvestment-related projects

Theory	Purpose	Context
Decision-making theory	To guide resource allocation decisions	Health service delivery organisations [16]
Deliberative democratic theory Deliberation theory	To capture stakeholder perspectives	Assisted Reproductive Technologies [15, 18] Pathology testing for vitamin B12 and folate [15]
	To underpin patient involvement	Priority setting healthcare improvement [13]
Social constructionist theory	To inform data analysis	Pathology testing for vitamin B12 and folate [15]
Resource allocation theory	To refine arguments in funding debate	Assisted Reproductive Technologies [14]
Prioritisation and quality improvement theories	To develop a proposal for rationalisation, prioritisation and rationing	Assisted Reproductive Technologies [17]

Perhaps the most relevant to disinvestment is the theory of discontinuance, defined by Rogers in his discussion of the theory of diffusion as *“a decision to reject an innovation after having previously adopted it”* [19]. In their review of diffusion of innovations in health care, Greenhalgh et al. note the importance of research into discontinuance and the lack of studies in this area [20]. Hollingworth et al. propose a schema of health technology adoption and withdrawal which includes both discontinuance and disinvestment [21] and Niven et al. use the definition of discontinuance for the term ‘de-adoption’ in their review of low-value clinical practices [22].

### Frameworks

Frameworks use concepts and relationships to provide a frame of reference, organise and focus thinking and assist interpretation. Frameworks are descriptive, tend to be high-level and can apply to a wide variety of situations [12, 23]. No frameworks for systematic, integrated, organisation-wide approaches to disinvestment were identified, however there are several frameworks for specific aspects of the disinvestment process. These are summarised by setting, aims, method of development and components in Table 3. Those applicable to the local healthcare setting are discussed in more detail under the relevant steps in the disinvestment process below.

**Table 3:** Examples of frameworks and models related to disinvestment

Framework/Model	Setting	Aims	Method of development	Components
PROJECTS TO IDENTIFY AND DISINVEST INDIVIDUAL TCPS				
Framework of potential settings and methods for disinvestment [5]	Organisation-wide program in local health service network	To identify potential settings and methods for disinvestment decision-making within local health service systems and processes	Literature review; survey of external experts, interviews and workshops with local stakeholders	Three organisational contexts that provide potential opportunities to introduce disinvestment decisions into health service systems and processes are presented in order of complexity, time to achieve outcomes and resources required: 1. Explicit consideration of potential disinvestment in routine decision-making for purchasing and procurement and development of guidelines and protocols, 2. Proactive decision-making about disinvestment driven by available evidence from published research and local data, 3. Specific exercises in priority setting and system redesign.
Algorithm for selecting a disinvestment project from a catalogue of potential opportunities [9]	Organisation-wide program in local health service network	To facilitate decision-making for identification of potential and selection of actual disinvestment projects	Literature reviews; surveys, interviews and workshops with local stakeholders; document analysis; consultation with experts; taxonomy development	Five steps in selection process: 1. Assess highest risk, 2. Assess importance and potential, 3. Assess quality and strength of evidence, 4. Assess extent of problem, 5. Assess implications of change. Three key decision-making steps between Steps 2 and 3, 3 and 4, and after 5. After selection: Notify decision; Implement; Evaluate; Report Each step includes the activities, who will undertake them, and the decision options
Model for an Evidence Dissemination Service [11]	Organisation-wide program in local health service network	To facilitate use of recently published synthesised evidence in organisational decision-making	Literature reviews; surveys, interviews and workshops with local stakeholders; document analysis; consultation with experts; taxonomy development	Methods and tools to identify sources of high quality synthesised evidence; automate methods of capture; classify, collate and store materials in useful categories; prioritise based on user and health service needs; repackage into suitable formats based on user needs; identify relevant individuals or groups to receive information; disseminate to the appropriate target groups, and report use of evidence
Guideline for Not Funding Health Technologies (GuNFT) [35]	Two versions are provided, one for application at national and regional level and the other at local level.	To facilitate establishment of a transparent, systematic and explicit process for assessing the potential for disinvestment in certain health technologies or in some of their indications	Literature review; face-to-face meeting, teleconference and emails using Nominal Group Technique with 10 experts representing health care delivery, administration, technology assessment and consumers to draft the guideline; validation by two external experts in HTA; wide circulation for comment and approval	Seven phases: 1. Identification through applications; 2. Validation of applications; 3. Prioritisation (if necessary); 4. Assessment; 5. Decision making; 6. Development of an action plan; 7. Diffusion of the decision, the reasons why it has been taken and the action plan. Applications are submitted by health care professionals; validation, prioritisation and assessment of the applications are undertaken by a HTA agency or the health service Technology Assessment Committee; and the decision, development of the action plan and diffusion is undertaken by the health service or regional health authority management team or other multidisciplinary body. Tools are available.
Disinvestment framework to guide resource allocation decisions in health service delivery [16]	Health service delivery organisations	To aid disinvestment activity in the local setting.	Thematic analysis of systematic review and a scoping review of the public sector and business literatures. Draft framework critiqued by Decision Maker Advisory Committee (Chief Financial Officers from Canadian health services) and External Reference Group (international academics) before being finalised.	Seven steps: 1. Determine objectives and scope; 2. Identify strategic priorities; 3. Identify options and risk; 4. Rank options; 5. Develop implementation plan; 6. Conduct disinvestment; 7. Assess outcomes and processes. Oversight Committee (senior managers and clinical leaders) is responsible for the majority of the process components including making final decisions; independent Assessment Committee (managers, clinicians, other staff and public representatives) defines the criteria, weights and scale used to assess disinvestment options, Support Committee (researchers and financial personnel) assists in the assessment of disinvestment options in the form of evidence, financial analysis and evaluative measures.
PROGRAMS FOR SECTOR-WIDE INVESTMENT AND DISINVESTMENT				
Framework of components in the resource allocation process [6]	Organisation-wide program in local health service network	To represent components in the process of resource allocation and the relationships between them	Interviews and workshops with stakeholders, thematic analysis of responses, document analysis, use of existing frameworks to synthesise findings	Eight components: Governance, Administration, Stakeholder engagement, Resources Decision Making, Implementation, Evaluation, and, when appropriate, Reinvestment. Details of elements of structure and practice within each component is provided. Structure is described as ‘who’ and ‘what’ and includes people, systems, policies, requirements, relationships and coordination. Practice addresses ‘how’ through processes, procedures, rules, methods, criteria and customs.
Model for Sustainability in Health care by Allocating Resources Effectively (SHARE) [8]	Organisation-wide program in local health service network	To develop, implement and evaluate organisation-wide systematic, transparent, accountable and evidence-based decision-making systems and processes	Three literature reviews; online survey, interviews and structured workshops with stakeholders; consultation with experts in disinvestment, health economics and health program evaluation; drafted in consultation with staff, consumers and external experts; assessed against framework for success and sustainability	Four components, each with multiple elements: 1. Systems and processes; 2. Disinvestment projects; 3. Support services; 4. Program evaluation and research. The model outlines each component and the relationships between them, their aims and activities as well as the underlying principles and the preconditions required for success and sustainability. There is also detailed discussion of the antecedents, barriers and enablers.
New Zealand National Health Committee Workplan [36]	National government decision-making	To provide the Minister of Health with recommendations for use and funding of health technologies	Not documented	The program addresses which technologies should be publicly funded, to what level and where technology should be provided and how new technology should be introduced and old technology removed. Six phases: 1. Identification, 2. Prioritisation, 3. Analyse and Assess, 4. Recommend, 5. Implement, 6. Evaluate.
Health technology reassessment and decommissioning framework/model [37]	National or provincial government decision-making	To create a model for assessing the health technology life cycle to identify and delist obsolete technologies	Focused narrative literature review and input from experts.	Two components: 1. Health technology life cycle and reassessment, 2. Reassessment and Decommissioning Model, with Oversight Committee, Triggers, and Possible Outcomes. Second component includes triggers and processes, structure (oversight committee), decisions and outcomes
PROGRAM EVALUATION				
Framework for evaluation of priority setting [39]	National, regional and individual healthcare facilities	To develop a framework for the evaluation of priority setting practice at macro and meso levels	Literature review and thematic analysis	Two evaluation domains: 1. Consequentialist outcomes: Efficiency, Equity, Stakeholder satisfaction, Stakeholder understanding, Shifted (reallocation of resources), Implementation of decisions, 2. Proceduralist conditions: Stakeholder engagement, Empowerment, Transparency, Revisions, Use of evidence, Enforcement, Community values
SHARE Program Evaluation Framework and Plan [8]	Organisation-wide program in local health service network	To assess the effectiveness of the SHARE program, implementation fidelity and factors for successful change	Drafts prepared by project team in consultation with Consultant in Health Program Evaluation to meet the information needs of key stakeholders and the internal capacity of staff conducting the project; revised and finalised in consultation with key stakeholders	Seven evaluation domains: 1. Improved patient care, 2. Improved resource allocation for health technologies and clinical practices, 3. Improved decision-making, 4. Improved staff capacity in use of evidence and data in decision-making and implementation of practice change, 5. Barriers and enablers, 6. Implementation fidelity, 7. Sustainability and spread. Includes an outcomes hierarchy based on the SHARE program components and a research program based on a theoretical framework for implementation of an evidence-based innovation.
Framework for evaluation and explication of the processes and outcomes of a disinvestment project [9]	Organisation-wide program in local health service network	To adapt a framework and taxonomy for evaluation of evidence-based innovations to enable evaluation and explication of disinvestment projects	Literature review, surveys and interviews with stakeholders	Three components: 1. Determinants of effectiveness (characteristics of external environment, organisation, proposal for change, rationale and motivation, potential adopters, potential patients, identification process, prioritisation and decision-making process, implementation plan, implementation resources); 2. Process of change (delivery of implementation strategy and stages of change); 3. Outcomes (process and impact for patient, practitioner, systems, economic, reinvestment, sustainability and spread). Taxonomy containing details within each component is provided.
Integrative framework for measuring overuse [38]	Relevant settings within health care systems	To assess the impact of efforts to reduce low-value care.	Not documented	Provides list of measurement tools linked to specific project/program goals and discusses advantages and disadvantages of each approach
STAKEHOLDER ENGAGEMENT				
SHARE model for incorporating consumer views into decisions for resource allocation [7]	Organisation-wide program in local health service network	To involve consumers in organisation-wide decision-making, capture their perspectives and incorporate them into decisions for resource allocation.	Literature review, individual and group interviews with Consumer Working Group and health service staff, workshop with Community Advisory Committee, drafting and revision with consumer participation.	Four components: 1. Principles, 2. Scope, 3. Preconditions, 4. Activities Activities include Consumer engagement (communication, consultation and participation) and use of Consumer evidence (consumer perspectives found in publications and data sources). Details of activities are reported in the context of the components of the resource allocation process noted above
New Zealand National Health Committee Workplan [36]	National government decision-making	To seek advice and engage with the health sector	Not documented	Tiered approach to engage with and seek advice from clinicians via colleges and specialty societies; providers such as District Health Boards, NGOs and private facilities via Health Sector Forum; international Health Technology Assessment agencies; Universities and Research Institutes, international and domestic manufacturers.

Polisena and colleagues [24] identified three frameworks in their review of disinvestment projects: Health Technology Assessment (HTA) [25], Accountability for Reasonableness (A4R) [26] and Program Budgeting and Marginal Analysis (PBMA) [27]. To distinguish between evaluation of new TCPs and those in current practice, the term Health Technology Reassessment (HTR) has been introduced for methods aiming to identify potential targets for disinvestment [28, 29]. HTA and A4R are frameworks by definition and are valuable tools for decision-making; however, although their use may lead to disinvestment, they are not frameworks specifically for disinvestment. Like A4R and HTA, PBMA and other priority setting frameworks [30–32] can play a key role in certain approaches to disinvestment, but do not address all potential aspects of the disinvestment process or all opportunities to drive change. However they would all integrate readily into a wider framework for disinvestment, as aspired to with the trialing of the Australian Medicare Benefits Schedule Review initiative [33]. Recently Elshaug et al. provided a comprehensive inventory of disinvestment policy and practice levers that could flow from HTA/HTR and other priority setting processes [34].

Sources of synthesised evidence such as HTAs, systematic reviews and evidence-based guidelines, can underpin disinvestment decisions in two ways. Firstly, the process of evidence synthesis can be undertaken reactively to address policy, management or clinical questions as they arise and inform the resultant decisions. Secondly, dissemination of the findings of published HTAs, systematic reviews or guidelines can be a proactive method of initiating decision-making to ensure policy and practice is consistent with the best available evidence.

The ‘Disinvestment framework to guide resource allocation decisions in health service delivery’ [16] and the ‘Guideline for Not Funding Health Technologies’ (GuNFT) [35] are examples of frameworks to identify and disinvest individual TCPs. They are very similar to the process outlined in the Workflow Diagram of the New Zealand National Health Committee for introduction of new and removal of old technologies [36]. All three are systematic, transparent and based on a series of steps to identify suitable TCPs, engage relevant stakeholders, make the appropriate decisions, implement and evaluate change.

The New Zealand National Health Committee also includes a framework for wider stakeholder engagement in their Business Plan [36].

Joshi and colleagues use both framework and model when referring to the outcome of their narrative review ‘Reassessment of Health Technologies: Obsolescence and Waste’ [37]. Based on the definitions used herein, it is classified as a framework. It includes the role of reassessment in the life cycle of a health technology and triggers, structures and outcomes for health technology reassessment and decommissioning.

Bhatia et al. present an ‘Integrative framework for measuring overuse’ as an evaluation tool to be implemented within initiatives that aim to reduce ‘low value care’ [38] and Barasa and colleagues propose a framework for evaluation of priority setting processes which considers both procedure aspects and outcomes in a range of contexts [39].

Conceptual frameworks developed in the SHARE Program for a range of purposes within the disinvestment process include potential settings and methods to integrate disinvestment into health service systems and processes [5], components in the resource allocation process [6], an evaluation framework and plan for the overall SHARE program [40] and an algorithm to facilitate decision-making for selecting projects from an evidence-based catalogue of potential opportunities for disinvestment [9]. An existing framework for evaluation and explication of implementation of an evidence-based innovation was adapted for use in disinvestment projects [9] and health information products and services [11].

### Models

Models are more precise and more prescriptive than frameworks. They are narrower in scope, the concepts are well defined and the relationships between them are specific. Models are representations of the real thing [12, 23].

The SHARE Program produced three models: integrating consumer values and preferences into decision-making for resource allocation in a local healthcare setting [7], exploring Sustainability in Health care by Allocating Resources Effectively in this context [8] and facilitating use of recently published synthesised evidence in organisational decision-making through an Evidence Dissemination Service [11]. These are summarised in Table 3. No other models for disinvestment were identified in the literature.

## New Framework

**Fig. 2:**
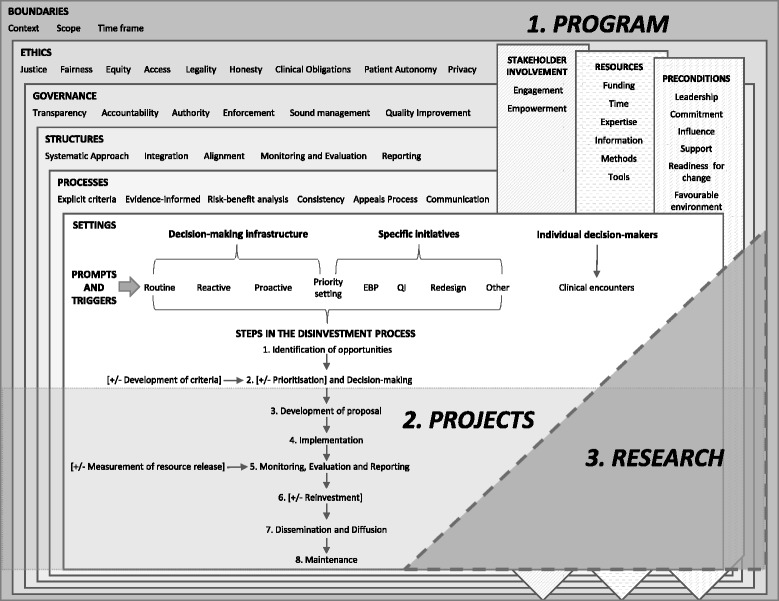
Framework for an organisation-wide approach to disinvestment in the local healthcare setting

Information pertaining to the practical and operational aspects of disinvestment in the local healthcare setting is presented and discussed in the context of a new framework (Fig. 2). The framework proposes a systematic approach that is integrated within organisational infrastructure. It brings together the definitions, concepts, principles, decision-making settings, potential prompts and triggers to consider disinvestment, and steps in the disinvestment process identified from the literature. It also seeks to remove barriers when it is possible to do so through establishment of new or adjustment of existing operational mechanisms. The details of each of the framework components are clearly articulated in the literature; many are derived from extensive work with stakeholder groups including decision-makers, policy-makers, health service staff, patients and members of the public.

The proposed framework builds on the work of others. While incorporating all the messages from the literature, it draws heavily on the three noted frameworks which identify steps in the disinvestment process [16, 35, 36]; the SHARE frameworks and models [5–9]; and other frameworks for introduction of new TCPs [2] and evidence-based change [41].

### Audience

The framework is aimed at health service decision-makers considering disinvestment and resource allocation, and health service researchers and implementation scientists working in this context.

The setting for this initiative was Monash Health, a large health service network in Melbourne Australia operating within a state-allocated fixed-budget model of financing. We anticipate results of this work and elements of the framework to have broader applicability and transferability, including to fee-for-service environments.

### Application

Decision-making in healthcare is described at three levels: macro (national, state/provincial and regional), meso (institutional) and micro (individuals) [42, 43].

The proposed framework was developed for use in policy, management and/or clinical decision-making at the meso level. It was designed to be embedded within existing systems and processes where it can be responsive to local needs and priorities at the level of implementation; for example health service networks, individual facilities, departments, wards or committees.

### Definitions

The lack of standardised terminology is a barrier to development of systematic approaches to operationalise disinvestment [1]. To address this, definitions and key concepts underpinning the framework are made explicit. The proposed framework provides a common language for researchers and decision-makers within and between programs, institutions and health systems making it easier to build and share a body of knowledge.

There are multiple definitions for disinvestment in the literature based on a range of different concepts [1, 44]. Numerous alternative terms conveying the same concepts are also in common use. Disinvestment is focused on the use of ‘health technologies’ but there is also a range of definitions for this term. To compound the difficulties arising from multiple definitions, the terms ‘disinvestment’ and ‘health technologies’ are frequently used in one way by researchers and in another by health service decision-makers [1]. Definitions relevant to the local healthcare setting are provided in Table 4.

**Table 4:** Definitions

Health technologies	Health products, devices and equipment used to deliver health care (eg prostheses, implantable devices, vaccines, pharmaceuticals, surgical instruments, telehealth, interactive IT and diagnostic tools). This is a narrow definition which reflects the common use by decision-makers and consumers in the local health care setting. Clinical practices, support systems, or organisational and managerial systems are NOT considered to be health technologies in this context.
Health technologies and clinical practices (TCPs)	Therapeutic, preventative and diagnostic procedures (eg use of products, devices and equipment PLUS medical, surgical, nursing, allied health and population health interventions). This is a pragmatic term to reflect the scope of most resource allocation decisions in the local healthcare setting.
Health programs and services	Agencies, facilities, institutions and the components within them that deliver health care, rehabilitation or population health practices such as health promotion and education.
Disinvestment	Removal, reduction or restriction of any aspect of the health system for any reason. Removal indicates complete cessation, reduction is a decrease in current volume or delivery sites, and restriction is narrowing of current indications or eligible populations. This is a broad definition, in essence the conceptual opposite of investment. This could apply equally to products, devices and equipment; clinical practices and procedures; health services and programs; information technology and corporate systems.
Principles	Fundamental qualities or elements that represent what is desirable or essential in a system.
Criteria	Standards against which alternatives can be judged in decision-making.
Routine decisions	Decisions made on a recurring basis or scheduled via a timetable eg annual budget setting processes, six-monthly practice audits, monthly Therapeutics Committee meetings, reviews of protocols at specified intervals after their introduction, etc.
Reactive decisions	Decisions made in response to situations as they arise eg new legislation, product alerts and recalls, applications for new drugs to be included in the formulary, critical incidents, emerging problems, etc.
Proactive decisions	Decisions driven by information that was actively sought for the purpose of healthcare improvement eg accessing newly published synthesised research evidence such as Cochrane reviews or Health Technology Assessments to compare against current practice, interrogating routinely-collected datasets to ascertain practices with high costs or high rates of adverse events, etc.
Prompt	An informal reminder or encouragement for thought or action.
Trigger	A formal mechanism that initiates or activates a reaction, process or chain of events.
Diffusion	Passive processes by which an innovation is communicated over time among members of a social system; usually unplanned, informal, untargeted, uncontrolled, decentralised, and largely horizontal or mediated by peers.
Dissemination	Active processes to spread knowledge or research eg publications, presentations and other deliberate strategies; planned, formal, often targeted, controlled or centralised, and likely to occur more through vertical hierarchies.
Maintenance	Active processes to sustain recently implemented change after project support is removed; to integrate the change into organisational systems, processes and practices; and to attain long-term viability of the change.
Methods and tools	Approaches, instruments or other resources that identify ‘what’ tasks are needed at each step and/or ‘how’ to undertake them. This is a pragmatic inclusive definition developed for use in this review to assist health service staff in disinvestment. This broad definition allows frameworks and models to be included if they meet these criteria.

We use the term disinvestment in the broadest sense, ‘removal, reduction or restriction of any aspect of the health system for any reason’. This can be applied to products, devices and equipment; clinical practices and procedures; health services and programs; information technology and corporate systems. Unlike most of the research definitions for disinvestment, this version is not constrained by a specified purpose (eg withdrawing practices of low value), defined criteria (eg effectiveness or cost-effectiveness) or anticipated outcome (eg reallocation of resources) which do not address cessation or limitation of TCPs for other purposes, based on other criteria, for different outcomes, which are likely to arise in local health services [1].

In contrast, we define health technologies in the narrowest sense; as products, devices and equipment used to deliver health care (eg prostheses, implantable devices, vaccines, pharmaceuticals, surgical instruments, telehealth, interactive IT and diagnostic tools) which reflects common use by health service decision-makers and consumers [1]. Clinical practices, health programs and services, information technologies, support systems, and organisational and managerial systems are not included in this definition. Although contained in many research definitions, they are not included in general references to health technologies in the local healthcare setting [1].

The terms ‘principles’ and ‘criteria’ are often used interchangeably; definitions for use in this review are included in Table 4.

### Concepts

The proposed framework is underpinned by several key concepts (Table 5). While disinvestment is the aim, it is not considered in isolation but in the context of resource allocation, addressing the spectrum of decision-making covering investment in new, continuation of existing, and disinvestment from current activities. The focus of the framework is positive: optimising healthcare, improving health outcomes, using resources effectively and efficiently. The components of the framework are integrated within current systems and processes and within existing health improvement paradigms such as evidence-based practice (EBP), quality improvement (QI) and system redesign.

**Table 5:** Concepts

Concept	Implication for framework
Use of the term disinvestment as a driver or justification for change is associated with negative connotations such as focusing on cost cutting, engendering suspicion and distrust, and getting stakeholders offside.	Do not use ‘disinvestment’ as the basis for the framework or the aim of change initiatives
Conducting disinvestment activities independently of existing systems and processes does not represent the reality of health service decision-making. It may be counterproductive: lacking incentives for change and introducing disincentives. Disinvestment should not be considered as an isolated activity, but integrated within existing systems and processes in the context of all resource allocation decisions, covering the spectrum from investment to disinvestment.	Implement disinvestment activities in the context of ‘resource allocation’
Removal or restriction of practices that are harmful or of little or no value; replacement of inferior practices with more effective or cost-effective alternatives; and reduction of organisational waste, systematic error and inappropriate use of TCPs all arise from good policy, management and clinical decisions. If these are based on evidence from research, local data and/or stakeholder views there are sound positive drivers for action. There is no need for the concept of disinvestment to be introduced as a reason for change.	Focus on the positive reasons driving removal, reduction or restriction of current practices Use existing systems, processes, expertise, methods and tools whenever possible
It has been proposed that disinvestment activities are more likely to be successful if decisions are transparent, integrated into everyday decision-making and central to local planning rather than ad hoc decisions, individuals ‘championing’ causes or standalone projects	
Disinvestment driven from a positive perspective focusing on optimisation of health care through allocation or reallocation of finite resources for maximum effectiveness and efficiency is more likely to be successful.	
Existing healthcare improvement paradigms such as Knowledge Translation, Evidence Based Practice, Quality Improvement, System Redesign and Health Economics offer theories, frameworks, methods and tools for decision-making, implementation and evaluation that can be applied to disinvestment.	

### Level of detail

Many of the elements within the proposed framework should be self-evident and be applied routinely as good practice, making it unnecessary to stipulate their requirement. However strong and consistent messages in the literature confirm that they are not standard practice and authors felt the need to state that they should be made explicit. Incorporating them all into a detailed framework achieves this.

Another reason for including all the elements in detail is to address potential ethical dilemmas [1]. In some circumstances it may be difficult to accommodate the principles of beneficence and utilitarian justice; clinicians advocate for the best interests of individual patients but resource allocation aims for the greatest benefit for the most people [45–47]. Similarly, arguments for equity may conflict with those for efficiency when the most efficient outcome is not the most equitable [48–50]. A systematic, transparent approach acknowledging these issues may facilitate difficult discussions and create potential for some efficiency to be traded away for equity maintenance or gain.

Some elements may be more important than others in individual situations. However, because they are all defined in the framework, the decision to exclude or reduce the role of some elements in extenuating circumstances becomes explicit. This strengthens the process and empowers those who have previously participated in suboptimal decision-making due to lack of resources, hidden agendas or organisational politics [6, 51–57].

### Components

The proposed framework is composed of three interconnected and interdependent components: 1) a program for organisation-wide decision-making, 2) projects to implement decisions and evaluate outcomes, and 3) research to understand and improve the program and project activities. Each component has a number of elements which are outlined in detail below.

### Characteristics

The framework is primarily descriptive to enable application in a local healthcare service and allow adaptation, replication and testing. It was developed using both deductive and inductive methods. Although not based on a specific theory, it has potential to facilitate future theory development and/or testing. Specific characteristics of the framework and potential for its use are summarised in Table 6 using domains and criteria developed to assess the robustness and utility of proposed models and frameworks [12]. This assessment enables potential users to identify whether the framework will meet their aims and be applicable to their situation.

**Table 6:** Characteristics of a framework for organisation-wide approach to disinvestment in the local setting

Domain	SHARE features
Purpose ▪ descriptive, explanatory or predictive	The framework is primarily descriptive to enable application and allow replication and testing. There are also some explanatory elements addressed in the relationships between components, for example ethical principles underpin all activities, decision-making settings sit within the scaffold of all eight principles, projects follow on from decisions, research is conducted in all aspects.
Development ▪ deductive or inductive ▪ supporting evidence	Methods used in development were both deductive and inductive. Evidence from research literature and other publications was the primary source. Many of these findings were based on extensive work with stakeholder groups. This was supplemented with experience from the SHARE program.
Theoretical underpinning ▪ explicit or implicit	No specific theory was used to underpin the framework.
Conceptual clarity ▪ well-described, coherent language for identification of elements ▪ strengths and weaknesses of theories ▪ potential to stimulate new theoretical developments	Three components are outlined in the framework: Program, Projects and Research. The Program is based on eight principles and nine settings for decision-making. The Projects are outlined in eight main steps. The relationships between them are captured in a diagram. Details of each component and the elements within them are provided in the text and in tables. No specific theories were used so no comparisons are made. There is potential for new theoretical developments if: ▪ specific theories are tested in development and implementation of the components ▪ components are removed or the relationships changed ▪ principles or pre-conditions are varied ▪ the framework is applied for purposes other than resource allocation ▪ the framework is applied in a range of contexts
Level ▪ individual, team, unit, organisation, policy	The framework was developed for implementation at meso level within the health system eg local network, institution, department, ward or committee.
Situation ▪ hypothetical, real	The framework represents actual settings and contexts in health service decision-making and implementation of change. However it could also be used for teaching or capacity building through hypothetical classroom discussions or simulation exercises.
Users ▪ nursing, medical, allied health, policy makers, multidisciplinary	The framework can be used by any decision-makers within the health system. While use of the framework could be initiated by any group, engagement and involvement of all relevant stakeholders is an underlying principle of application. The framework could be used in policy, management or clinical contexts.
Function ▪ barrier analysis ▪ intervention development ▪ selection of outcome measures ▪ process evaluation	The main function is to establish and maintain systems and processes to make, implement and evaluate decisions regarding resource allocation and research the components involved. The principle of evidence-based implementation requires assessment of barriers and enablers but the framework itself does not specifically facilitate this process other than to prompt users. Details of barriers identified from the literature are contained in the text and tables. The steps within the Project component will facilitate development of an intervention for systematic evidence-based decision-making and implementation of change. Evaluation of process and outcomes is a key element; however selection of variables and outcome measures is not facilitated by the framework per se, other than to prompt users to take an evidence-based approach. Examples of measures proposed by others are included in the text.
Testable ▪ hypothesis generation ▪ supported by empirical data ▪ suitable for different methodologies	The framework describes principles to underpin robust decision-making, settings and opportunities, implementation of change and evaluation of process and outcomes. A range of hypotheses could be developed for each of these elements and the relationships between them which could be tested in a number of ways using various methodologies. The framework could also be tested beyond the local healthcare level, at national or state/provincial level; or outside the health context in education, community development, social services, etc

## Program

### Principles for decision-making

Forty-two principles were identified from the existing literature and the SHARE publications and grouped into eight categories that emerged from these findings: Boundaries, Ethics, Governance, Structures, Processes, Stakeholder involvement, Resources and Preconditions. These are presented in the framework as two groups (Fig. 2).

The first group have a hierarchical relationship depicted as a series of nested boxes. The whole program is defined by explicit boundaries, ethical principles underpin good governance, governance directs and controls structure, and structure enables and accommodates process. The decision-making settings, prompts and triggers all sit within the scaffold of these five categories.

The second group, represented as three vertical bars, are required across all of the other elements. For example, stakeholders need to be involved in defining the boundaries and establishing the ethical parameters and methods of governance; they should be included in the structures and processes and participate in the projects and research. Adequate and appropriate resources and the noted preconditions will be required to establish, maintain and improve all aspects of the framework.

The intersection of the two groups of principles also demonstrates that ethics, governance, structures and processes also apply to stakeholder engagement, resources and preconditions. For example, stakeholder engagement should be systematic and integrated, funding should be sourced ethically and influence should be transparent.

These principles and their relationships also apply to the project and research components.

Further details of the categories, full descriptions of individual principles, and related citations are outlined in Additional file 1.

### Settings

Nine settings for decision-making are described in three categories: Decision-making infrastructure, Specific initiatives and Individual decision-makers.

While the framework is proposed for organisation-wide application, any of the nine settings could be considered individually. A framework for a single setting would be underpinned by the same principles, decisions would lead to projects with the same steps and research could be conducted on all elements.

#### Decision-making infrastructure

Each sector of the health system has an organisational infrastructure of decision-making settings where committees, designated panels or individuals with delegated authority make decisions on behalf of the jurisdiction or individual facility. A classification system and descriptors for decision-making settings, decision-makers, scope and type of decisions in the local health service setting was developed in the SHARE Program [6].

Decisions can be categorised as routine, reactive and proactive [6, 58]. Routine decisions are made on a regular basis; reactive decisions are made in response to situations as they arise; and proactive decisions are driven by information that was actively sought for the purpose of healthcare improvement. Examples are included in Table 4.

A range of potential decision-making activities are outlined in Table 7 [1, 5, 6, 8, 59–61]. Most of these occur in more than one of the three categories of decision-making and can be used for more than one aspect of the disinvestment process. Development or revision of guidance documents is a good example. Guideline and protocol development can occur routinely, particularly when existing documents are updated at regular intervals; in reactive situations such as a critical incident which highlights lack of guidance in a specific area; or when proactive use of research identifies that current documents do not reflect the best available evidence. Disinvestment opportunities can be identified if the systematic review process undertaken when initiating or revising a guidance document determines that a TCP, service or program should be removed or replaced [5, 17, 60–63]. Guidance documents can also be used to implement disinvestment decisions and audit of guideline adherence can measure the results [59, 60, 64–66]. Manuals for guideline or protocol production could include prompts to note and follow up opportunities for disinvestment as part of the document development process [5].

**Table 7:** Examples of activities and settings for disinvestment within decision-making infrastructure

Activity	Example	Routine	Reactive	Proactive	Priority Setting
Meeting external requirements	▪ Addressing legislative, regulatory and accreditation requirements, national and professional standards, etc	✓	✓		
	▪ Responding to product alerts and recalls		✓		
Setting budgets	▪ Determining sources of income and items of expenditure	✓			✓
Spending money	▪ Introducing new items to funding lists. Examples include, but are not limited to, national health schemes, insurance benefits schedules, institutional lists of permitted TCPs, formularies.	✓	✓	✓	✓
	▪ Commissioning health services and programs	✓	✓	✓	✓
	▪ Procuring capital works, plant and equipment	✓	✓	✓	✓
	▪ Purchasing clinical consumables	✓	✓	✓	✓
	▪ Assessing grant and funding applications	✓	✓		
Allocating non-monetary resources	▪ Allocating people, time, access to facilities, etc	✓	✓	✓	✓
	▪ Developing guidance documents, promotional information or educational materials that indirectly allocate resources. Examples include, but are not limited to, peak body recommendations, clinical guidelines, protocols, standard operating procedures, decision support systems, posters, presentations.	✓	✓	✓	✓
Making strategic and operational decisions	▪ Developing goals and strategies for Strategic Plans	✓			✓
	▪ Developing outcomes measures and targets for Business Plans	✓			✓
Using evidence to initiate and/or inform decisions	▪ Updating existing evidence, undertaking Health Technology Reassessment, etc.	✓	✓	✓	
	▪ Accessing and utilising research evidence, population health data, local health service data, consumer and staff feedback	✓	✓	✓	✓
Evaluating outcomes of previous decisions and projects	▪ Monitoring, evaluating and reporting of all newly introduced TCPs to see if they perform as expected, post marketing surveillance	✓			
	▪ Monitoring, evaluating and reporting of purposive or random samples of decisions	✓	✓	✓	
	▪ Monitoring, evaluating and reporting of purposive or random samples of projects	✓	✓	✓	

Formal priority setting exercises may also be built into the decision-making infrastructure. These determine which TCPs, programs or services to introduce, maintain or remove based on a pre-determined set of criteria. An example might be annual capital expenditure decisions. In this situation, priority setting could be classified as ‘routine’, however it is noted separately in the framework as it also commonly arises in the context of individual initiatives described below.

#### Specific initiatives

In addition to the decision-making settings outlined, specific initiatives to improve practice are undertaken by health services, many of which involve disinvestment. These may be instigated by government, management or health practitioners, and although there is considerable diversity, most are related to EBP, QI, system redesign or economic approaches to priority setting such as PBMA [1, 6, 34]. Some projects may set out to disinvest, others may have quite different initial aims but the need for disinvestment becomes apparent during the project.

An EBP approach might be to remove or reduce use of inferior practices identified from systematic reviews, HTAs, evidence-based guidelines or ‘low value’ lists, or reduce their use to levels deemed clinically appropriate [9]. Clinical audit, QI and system redesign methods may be used to tackle inappropriate use of TCPs or organisational waste. Priority setting exercises like PBMA consider the costs and benefits of relevant alternatives in an aspect of healthcare delivery to determine the maximum outcome from the available resources.

There are several examples of disinvestment-related initiatives with relevance at the local health service level. Therapeutic equivalence or drug substitution programs involving replacement of expensive drugs with equally effective but lower cost alternatives from the same drug family has demonstrated considerable cost saving in macro and meso programs [67, 68]. Generic prescribing, substituting brand name drugs with generic alternatives, has been addressed at international, national, institutional and individual levels with mixed outcomes [69–72]. Benchmarking the results from individual interventions or programs across different health providers aims to ascertain best practice which others can aspire to and which can be applied at all levels; but by simultaneously identifying inferior practices it can also be used as “*a tool to start a disinvestment dialogue*” [21, 73, 74].

#### Individual decision-makers

At the micro level, the term ‘disinvestment’ is not generally applied to changes initiated by individuals; however the principle is the same. Individuals cease or restrict practices when they become aware of new evidence or to address local needs and priorities.

Much of the literature on decision-making focuses on how money is spent, however there are considerable opportunities for disinvestment in allocation of non-monetary resources. Although clinical encounters do not usually involve funding decisions, they offer opportunities to consider disinvestment in use of other resources such as ordering tests, referring to other practitioners, using drugs and other therapies, or undertaking procedures. An example is the Choosing Wisely program being replicated in national campaigns across the world which highlights potentially ‘low value’ treatments and tests so that clinicians and consumers can consider the relative benefits in their specific situations [75].

### Prompts and triggers

Prompts and triggers are proposed to initiate and facilitate identification of disinvestment opportunities. Prompts are informal reminders or encouragement for thought or action and triggers are formal mechanisms that initiate or activate a reaction, process or chain of events (Table 4). The settings above provide opportunities to introduce systematic prompts and triggers to use evidence from research, data and stakeholder feedback to drive decision-making.

Prompts, triggers and potentially even mandatory requirements to consider disinvestment could be built into existing decision-making infrastructure [5, 37]. Using expenditure decisions as an example, prompts and triggers could be incorporated into meeting agendas of finance committees, budgeting processes, application forms, algorithms, protocols or checklists. Mandatory requirements to consider disinvestment could be implemented as specific directions within purchase orders, explicit decision-making criteria for committees, or steps in application processes that require authorisation. Additional examples of prompts and triggers at the organisational level are outlined in Table 8.

In specific initiatives to implement health service improvements, prompts and triggers to consider disinvestment could be introduced into project management templates or training programs for project management, change management, quality improvement processes, etc.

Prompts, triggers and mandatory requirements could also be used to guide the decisions of individual practitioners in clinical encounters; these could be included in local guidelines and protocols to steer practice away from unsafe, ineffective or inefficient use of TCPs.

**Table 8:** Examples of systematic prompts and triggers to initiate disinvestment decisions

▪ Approve introduction or continuation of TCPs for limited time only and require review of desired outcomes, costs, etc. before re-approval is granted at end of time period
▪ Approve new guidelines and protocols for limited time only and require review of evidence, costs, etc. and appropriate revision before re-approval is granted at end of time period
▪ Include steps that consider disinvestment of existing practices in manuals for guideline and protocol development
▪ Include steps that consider disinvestment of existing practices in checklists for a range of organisational decisions
▪ Add consideration of disinvestment to templates for meeting agendas where appropriate
▪ Mandate consideration of disinvestment in procurement processes: include in requistion documents and require sign off by relevant body overseeing disinvestment at appropriate level
▪ Systematically ascertain evidence from research, data or stakeholder feedback, send directly to decision-makers and seek and/or require response
▪ Incorporate flags and/or question use of low value TCPs in clinical decision support systems
▪ Build questions about potential disinvestment into business case templates and application forms for grants, changes to formulary, introduction of new TCPs, etc.
▪ Introduce requirements for consideration of disinvestment into documents governing scope of decisions such as position descriptions and committee Terms of Reference
▪ Add prompts to consider disinvestment to data reports, scorecards, dashboards, etc.
▪ Add prompts to consider disinvestment in project management templates and training programs for project management, change management, quality improvement processes, etc.
▪ Build disinvestment into strategic planning processes
▪ Build disinvestment KPIs into business plans or performance plans
▪ Consider ‘one for one’ swaps where a new TCP can only be introduced if an old one is removed

### Steps in the disinvestment process

The disinvestment process begins when opportunities for disinvestment are identified from the activities in the settings above. Eight steps in the disinvestment process were ascertained from existing frameworks [6, 16, 35, 36]: Identification of opportunities; Prioritisation (if required) and Decision-making; Development of a proposal; Implementation; Monitoring, Evaluation and Reporting; Reinvestment (if required); Dissemination and Diffusion; and Maintenance. Two additional elements are included: some projects may require development of local criteria for prioritisation and decision-making and projects that aim to reinvest will need to measure the resources released as part of the evaluation process.

The first two steps are part of the decision-making program, the following six are undertaken in projects arising from the decisions.

## Projects

Once a decision has been made, a project to implement it can be initiated. While individual projects will have specific characteristics and requirements such as aims, objectives, timelines, budgets, deliverables, roles and responsibilities, the principles outlined in the framework apply to all project activities.

Examples of methods and tools for disinvestment are discussed below; however the proposed framework does not stipulate project design or conduct, allowing application of any theories, methods or tools at each step.

## Research

Research is required to understand and improve the program and project activities. It is overlaid across all elements in the diagram to represent the potential for research in each aspect of the framework.

## Methods and tools

There are many definitions for the terms theory, framework, model, method, tool, strategy and related concepts. Some definitions note specific features that make the terms mutually exclusive, others allow the terms to be used interchangeably, and some overlap. In this review, the label ‘methods and tools’ is used pragmatically to assist health service staff in disinvestment and includes approaches, instruments or other resources that identify ‘what’ tasks are needed at each step and/or ‘how’ to undertake them. This broad definition allows frameworks and models to be included if they meet these criteria.

Appropriate, valid and reliable methods and tools are required for effective decision-making, implementation and evaluation. The resources identified are described briefly but no evaluation was undertaken due to lack of relevant data; some have been piloted and refined, but most have no published reports of their effectiveness or impact. The availability of validated materials is noted where appropriate. Hence users will need to consider the validity and applicability of these resources in their individual contexts.

There are many sources of generic advice for ascertaining and utilising evidence, undertaking and applying health economic analyses, making decisions, implementing change and evaluating outcomes including, but not limited to, The Cochrane Library, Canadian National Coordinating Centre for Methods and Tools, UK National Institute for Health and Care Excellence (NICE), US Institute for Healthcare Improvement, US Centers for Disease Control and Prevention, and US Agency for Healthcare Research and Quality.

There are also many methods and tools from other areas of health research and practice that are relevant to disinvestment which could be employed within this framework; knowledge translation, EBP, QI, system redesign and other improvement methodologies all have well-developed validated processes that are familiar to health service staff [1]. While there are few published examples of successful initiatives labelled as ‘disinvestment’ within local health services, there are many examples in the EBP and quality and safety literature of disinvestment-type activities where TCPs that are unsafe or ineffective have been discontinued. A review of ‘de-adoption’ summarises 39 such interventions that provide information on several steps in the disinvestment process [22].

Two publications provide advice in a range of areas relevant to disinvestment. A book on rationing, priority setting and resource allocation in health care discusses multiple generic and specific methods and tools suitable for disinvestment including stakeholder participation, leadership, economic evaluation and several of the steps in the disinvestment process [76]. A toolkit for decommissioning and disinvestment, defined as withdrawal of funding from the provider organisation, provides high-level guidance on governance and administrative matters for removal of health services, not individual TCPs, and some tools for assessing service performance against UK data [77].

The GuNFT guideline provides guidance on establishment of a decision-making program and recommendations, templates and other tools for several steps in the disinvestment process [78]

Several products from the SHARE Program also address a range of principles and steps in the disinvestment process.

- Summaries of issues to consider in development of an organisational program for disinvestment [5] and implications for disinvestment in the local setting [8] were compiled.
- An investigation of the resource allocation process in a local health service generated a framework of eight components, the relationships between them, and features of structure and practice for each component [6]. Structure is described as ‘who’ and ‘what’ and includes people, systems, policies, requirements, relationships and coordination. Practice addresses ‘how’ through processes, procedures, rules, methods, criteria and customs.
- A classification of decision-making settings, decision-makers, and scope and type of decisions was developed and strengths, weaknesses, barriers and enablers to resource allocation in a local health service were ascertained [6].
- A model for exploring Sustainability in Health care by Allocating Resources Effectively (SHARE) in the local healthcare setting brings together systems and processes for decision-making; identifying and undertaking disinvestment projects; support services to facilitate making, implementing and evaluating decisions; evaluation and research to measure and understand the processes and outcomes of these disinvestment-related activities; and principles and preconditions for success and sustainability [8].

Methods and tools for the principles are presented in Additional file 1.

### 1. Identification of opportunities

Potential disinvestment opportunities can be derived from all of the decision-making settings discussed above, either incidentally or systematically from prompts or triggers embedded in local systems and processes. However, at the health service level, it is more common for disinvestment opportunities to be identified through ad hoc proposals based on individual’s observations or local knowledge than through a systematic evidence-based approach [9, 21, 79, 80].

The sources of information noted in the literature that could be used in these settings to identify disinvestment opportunities include research, health service data, expert opinion and stakeholder consultation. While any one of these sources could identify a potential target for disinvestment, ideally information from all four would be combined in confirming the appropriateness of the choice [5]. Evidence from research would be considered in light of local data. For example, if a systematic review or HTA identified a more cost-effective intervention to one in current use, decision-makers could use local data to assess whether the burden of disease, volume of use, likely impact and potential cost of change warrant the required disinvestment activities. Similarly, evidence from local data would be enhanced by using the literature to identify best practice. For example, if an audit of prescribing rates of a high cost drug finds variation between departments, a review of the appropriate research would confirm whether the higher rate is overuse and should be reduced or the lower rate is underuse and should be increased. Expert opinion and stakeholder consultation add clarification and important perspectives to these decisions and may also reveal examples of inappropriate use of TCPs not identified by other methods. The SHARE Program used the SEAchange model [41], a formal evidence-based approach to change, to ensure that evidence from research and local data, experience and expertise of health service staff, and values and perspectives of consumers were considered at each step (Fig. 1) [3].

#### 1.1 Research

Reactive decisions can be informed by synthesised evidence and relevant primary studies; the type of research design and level of evidence required depends on the context of the decision and the nature of the question being addressed. Rigorous evaluation of new TCPs prior to inclusion in nationally funded health schemes has been standard practice for the past two decades and high quality HTAs, systematic reviews, evidence based guidelines and clinical effectiveness research reports have been developed to determine other national health policies. There is also a long history of locally-developed HTAs for use in decisions about introduction of new TCPs at health service level [2, 81]. Health technology reassessment of existing TCPs with view to identifying potential targets for disinvestment has been undertaken at both national and local level [28, 29, 82, 83].

Systematic use of research in routine decisions is evident in reassessment of new TCPs at specified time periods after their introduction at national [72, 84] and local level [2]. At the other end of the TCP lifespan, *“obsolescence forecasting”* has also been proposed as a systematic approach to initiate HTR when it is anticipated that *“a new, more functional product or technology supersedes the old or when the cost of maintenance or repair of old technology outpaces the benefits of a new piece of technology”* [37].

Examples of proactive use of research for disinvestment at national level include a review of all listed drugs conducted in France resulting in removal of 525 drugs considered to have *“insufficient medical value”* [72] and commissioning of a complete review of the Australian Medicare Benefits Schedule (fee-for-service) to ensure that all funded items are safe, effective and cost-effective [33]. There are other examples of systematic and ad hoc use of research to drive disinvestment at national level [60, 72, 85].

Similar approaches have been used at local level where organisations have reassessed all of the TCPs related to a specific clinical issue or area, or reassessed one particular TCP at a time [83]. The SHARE Program implemented an Evidence Dissemination Service to proactively retrieve, appraise, summarise and categorise synthesised evidence from high-quality sources soon after publication and deliver it directly to the relevant designated groups and individuals responsible for organisational decision-making related to resource allocation [11]. The SHARE Program also proposed a framework for consumer involvement that included proactive use of sources of published consumer evidence [7].

Lessons from these national and local examples may be useful to those undertaking local disinvestment initiatives.

High quality sources of research evidence are available and readily accessible through online resources, however there are some challenges to their use in the local health service setting.

Health service staff report lack of time, knowledge, skills and resources as barriers to searching for, accessing and appraising research; and that evidence is not used systematically or proactively to inform decisions [6, 10, 86–96]. Reports of HTAs undertaken by local health services [81, 97] and decision-making for use of TCPs [2, 98–100] note limitations in local processes, resources and expertise resulting in decision-making with varying degrees of rigour, structure and transparency. In addition to expertise, training and support, systematic prompts and triggers to use research evidence in all three types of decision-making are needed at the local level and could also be used to identify relevant TCPs for disinvestment or initiate discussions on potential disinvestment topics.

There are also limitations in coverage and applicability of currently available synthesised evidence to address all the needs of local decision-makers. The topics reviewed by national agencies are most frequently medical interventions, pharmaceuticals and diagnostic tests that have a high profile and are expensive as individual items. While these are obviously important in local health services, lower profile areas such as nursing and allied health practices, service delivery options, models of care and clinical consumable items, all of which have potential for considerable improvement in patient outcomes and reduction in costs and resource utilisation, are less commonly addressed in these formats, leading to locally-conducted HTA/HTR with the shortcomings noted above.

These limitations have additional implications for local health services given the lack of standardised methods for HTR [37, 82, 83]. Further research in this area has been proposed to develop consistent methods which will increase rigour, enable replication, enable comparison with others, facilitate application in equivalent situations to reduce duplication, engender familiarity and understanding to increase uptake and use of content, and build on existing work [28, 29, 83].

#### 1.2 Health service data

Routine, reactive or proactive investigation of available data can identify potential opportunities for disinvestment. There are many generic tools like dashboards, statistical process control or balanced scorecards available to analyse health service data, however none were identified in this review of the disinvestment literature. These tools, plus simple data interrogation methods, can identify factors associated with TCPs that might be worthy of further exploration as candidates for disinvestment; for example high volume, high cost, long length of stay and high rates of mortality, adverse events, readmission or reoperation, and geographic variation [5].

Searching routinely-collected datasets for known ‘low value’ practices is a direct and potentially productive method of identifying disinvestment opportunities [38, 101, 102]. With initiatives such as Choosing Wisely proliferating, it is now less a case of list-making as list-taking and prioritising. An algorithm developed in the SHARE Program for selection and prioritisation of disinvestment projects from a catalogue of potential targets derived from the research literature using locally-developed criteria could be adapted for use with a collection of potential targets identified from investigation of local data [9].

There is a large body of literature on examination of practice variation [103]. Reporting on variations in healthcare practice has been done at national and regional levels and atlases of healthcare variation have been produced [104–108]. Similar processes could be undertaken at local level. Comparisons can be made between regions, facilities, departments and individual practitioners, or over time; but should only be done when the population demographics, socio-economic factors and particularly patient acuity are similar [5, 21, 73, 105, 109, 110].

Recent studies have investigated practice variation specifically to identify ineffective practices; they note the potential to do so within local health services or for health services to benchmark against their counterparts [21, 105, 110]. Examination of health service utilisation and patient outcomes data, as well as differences in rates of prescribing, ordering diagnostic tests or use of specific interventions, could indicate inappropriate or suboptimal practices suitable for disinvestment. Procedures with high variability are often not on the ‘low value’ lists, indicating additional possibilities to identify disinvestment opportunities from this approach [21].

Use of local data clearly has potential but problems with data validity, reliability, comprehensiveness and degree of sensitivity to disinvestment requirements remain significant barriers [21, 24, 46, 48, 58, 60].

There are many methods for analysis, synthesis and interpretation of data however, like research evidence, there is a lack of systematic prompts or triggers to use them [5, 21]. While not specifically directed at disinvestment or resource allocation, a conceptual framework and logic model developed by Nutley and colleagues for improving data use in health system decision-making could facilitate a more proactive, systematic approach [111, 112].

The aims of the SHARE Data Service were 1) to interrogate routinely-collected data to identify potential disinvestment opportunities and communicate this information to appropriate decision-makers and 2) to respond to requests from decision-makers to assess local data related to potential disinvestment opportunities that had been identified from the research literature [10]. Although the Data Service was not implemented due to unanticipated local factors, the decisions underpinning the design and the models proposed may be helpful to local health services wishing to establish similar resources to support use of data in the disinvestment process.

#### 1.3 Stakeholder nominations

Stakeholder engagement is noted as a fundamental principle of the decision-making process and involvement of stakeholders and local ownership of decisions and projects are noted as facilitators of change in general [113–115] and in relation to disinvestment [21, 58, 72, 82].

The Ontario Reassessment Framework gives priority to potential candidates for disinvestment if nominated by a clinical expert [85] and four frameworks for disinvestment employ applications from stakeholders in the identification process [9, 16, 35, 36].

Participants in the SHARE Program noted that, while formal prompts and triggers could be built into existing decision-making infrastructure, there are also informal yet systematic approaches that could be integrated into other systems and processes to facilitate identification of opportunities for disinvestment by health service staff [9]. Examples are included in Table 9.

**Table 9:** Additional systematic methods to facilitate identification of disinvestment opportunities in a local health service

▪ Discuss principles of disinvestment and examples of successful projects at department/unit meetings, educational events, etc.
▪ Assign a group member to look for disinvestment opportunities in committee/working party decisions
▪ Add a disinvestment question to the ‘Leadership Walkround’ protocol
▪ Identify clinical champions interested in disinvestment in each program/department/unit who would look out for opportunities
▪ Support staff who have undertaken a disinvestment project to look for more opportunities
▪ Have disinvestment as a high priority in medication safety reviews
▪ Encourage or require projects that are introducing something new to have a component of disinvestment
▪ Review projects that are being conducted for other reasons and identify and focus on any disinvestment elements

Stakeholder nomination can be a powerful contribution to the process, providing the nominated items are objectively scrutinised against additional identification and prioritisation criteria [109], however there are some considerations in the actual implementation.

Although evaluation of the applications in these frameworks is rigorous, based on explicit local criteria and health technology assessment, the process of how the topic was raised initially is not systematic or transparent. Applications can be received from any stakeholder for any reason. In this context they are likely to be driven by non-systematic factors such as clinician’s interests, information obtained from conferences or journal articles, or awareness of practice elsewhere [2, 6]. *“Understanding how the technology got on the agenda, where it came from and who was pushing for it”* and the potential for *“gaming by industry”* are concerns reported by senior health decision-makers [116], but are often unclear in a stakeholder application process.

When invited to nominate candidates for disinvestment, clinicians have been found to be more likely to identify the practices of other professional groups than their own, practices that do not affect their revenue-generating services and practices of low impact [9, 21, 117].

Clarity of aims and objectives at the start of a project and clear rationale for change were in the top 10 considerations for successful disinvestment and one of three best practice recommendations arising from a Delphi study of international experts [52]. However lack of clarity and rationale have been noted as problems in identifying disinvestment opportunities [63], particularly from stakeholder applications [9, 10].

These issues may create systematic biases in the choice of investment targets and miss some key opportunities. Unnecessary duplication of effort may also result, with individual facilities or regions undertaking extensive evaluations of the same topics.

#### 1.4 ‘Low value’ lists

‘Low value’ lists are compilations of practices that have been demonstrated to have little or no benefit or potential to cause harm. They have been developed by governments and health agencies [118–120], commissioners of health services [121], professional bodies [65, 122, 123] and researchers [124–126]. Some of these lists are derived from research evidence, some are based on expert opinion and others from a combination of the two.

Duckett and colleagues separate them into ‘top down’ and ‘bottom up’ approaches, noting that each has benefits and drawbacks [73]. The ‘top down’ approaches, such as the UK National Institute for Health and Clinical Excellence ‘Do Not Do’ Recommendations [54], are described as providing the most consistent, objective, transparent and relevant evaluations. The ‘bottom up’ approaches, such as the Choosing Wisely program [74], highlight potentially ‘low value’ treatments and tests that should be carefully considered at the point of care.

Removing, reducing or restricting practices of little or no value clearly has merit, and ‘low value’ lists are likely to be very useful to health service decision-makers if they are based on sound evidence backed by expert consensus. However the definition of ‘low value’ is not always explicit and the validity and appropriateness of some of the lists and the ethics of their application have been questioned [117, 125, 127–130]. Potential users of ‘low value’ lists may wish to confirm the basis for claims made, in particular the definition being used and the use of systematic review evidence in the inclusion process [9].

The SHARE algorithm described earlier could also be applied to ‘low value’ lists to assess local applicability and facilitate prioritisation [9].

#### 1.5 Economic approaches to priority setting

These priority setting approaches combine evidence from local data, expert opinion and stakeholder consultation [27, 32].

PBMA applies the economic principles of opportunity cost and marginal analysis to determine priorities for health program budgets in the context of limited resources [131]. This method approaches disinvestment from the relative perspective, with decision-makers weighing up options for investment and disinvestment and reaching their preferred balance using locally-relevant criteria established by the stakeholders. The process is well-tested and guidance is available [27]. Although decision-makers acknowledge the usefulness of PBMA, it remains quite difficult to achieve in practice [24, 48, 131]. Another criticism is that it fragments the health sector into ‘program budget silos’ resulting in allocation and re-allocation of resources within, rather than between, programs which fails to identify more cost-effective options outside the program area [31, 48, 131, 132].

In contrast to PBMA, the Health-sector Wide model is designed to shift the focus of priority setting away from program budgets towards well-defined target populations with particular health problems [31]. The condition-specific silos created here may be an improvement on program budget silos, but the model is more difficult to apply in local health services where funding decisions are not based on condition-specific populations.

The major limitations for all priority setting approaches include idiosyncrasies in cost-accounting, lack of sufficient high quality data to inform decision-making, and lack of time and appropriate skills of decision-makers to undertake the process and implement the decisions [24, 27, 46, 48, 55, 131]. Lack of in-house expertise in health economics is a particular barrier at the local level [9].

### 2. Prioritisation and decision-making

Priority setting exercises clearly include a prioritisation process, however initiatives that identify their disinvestment targets by other means may need a specific prioritisation process to choose between the options available. Methods and tools for systematic, transparent and equitable decision-making may be used if prioritisation is not required or to complement the prioritisation process.

Prioritisation tools primarily focus on characteristics intrinsic to the TCP; however additional criteria may influence the decision to proceed with a disinvestment project in the local healthcare setting [9]. These might be pragmatic features that enhance initiatives chosen specifically as pilot or demonstration projects, such as opportunities for ‘quick wins’, or factors that affect the outcome of a project, such as likelihood of success and sustainability or potential usefulness of the evaluation.

There is a huge range of potentially relevant criteria for resource allocation decisions. Most authors emphasise that a list of criteria should be developed with input from all stakeholders to meet the objectives of individual situations. The commonly cited basic requirements include clinical parameters such as safety and effectiveness, economic measures such as cost-effectiveness and affordability, and social factors such as local values and priorities. Additional criteria will depend on the setting and context. Methods and tools to assist in assessment of safety and effectiveness [133–136] and use of economic measures [137–139] are available. Similar resources for consumer and community engagement are addressed in Additional file 1.

Deciding between several alternatives is a complex process requiring consideration of multiple factors. Health service decision-makers are often not good at this, relying on heuristic or intuitive approaches which ignore potentially important information [140]. Methods such as burden of disease analyses, cost-effectiveness analyses and equity analyses focus on some but not all of the available information [140]. Multi-criteria decision analysis (MCDA) allows consideration of all factors simultaneously, and although used widely in other scientific disciplines, it has only been used in health care relatively recently [76, 140].

The Star model, a “*socio-technical allocation of resources*” based on MCDA and health economic theory, has been piloted successfully in two settings, revised and developed into a toolkit [141–143]. MCDA is also the foundation of the Evidence and Value: Impact on DEcision Making (EVIDEM) framework, which is being investigated further through research conducted by the international EVIDEM Collaboration [144].

While the components of the A4R framework are included within several principles in the new framework, policy makers, managers and clinicians may also wish to use the A4R terminology specifically in their decision-making processes.

A4R is also the basis for the Systematic Tool for Evaluating Pharmaceutical Products for Public Funding Decisions (6-STEPPPs) [145] and A4R and MCDA have been combined in other decision-making applications [146, 147].

Lists of criteria for consideration in prioritisation and decision-making have been published for disinvestment [78, 82, 85, 109, 148], including many who have applied or adapted the criteria framework proposed by Elshaug et al. [72]; resource allocation [6, 149–151]; and general decision-making [42]. A tool to analyse gaps in priority setting has also been developed [152].

Many health service decision-makers use a prioritisation matrix, but most of these are developed locally and based on simple spreadsheets or business case templates [9, 48, 55, 153]. This variety of tools makes it difficult to compare costs and outcomes more broadly and there is some scepticism amongst decision-makers about the lack of rigour, transparency and skills involved in their local programs [21, 48].

There are also software applications to facilitate PBMA and generic prioritisation processes [27, 154, 155].

### 3. Development of a proposal

Once a decision has been made that there is a need for change, a proposal to meet that need and implement the decision is developed. When the proposal is drafted, the time and other resources required to implement and evaluate it can be assessed to determine if the benefits outweigh the costs of the exercise and to inform planning.

The range of potential disinvestment activities is broad and disparate. A proposal to remove a drug from a hospital formulary is likely to be very different to a proposal to close down an inpatient facility. No specific methods and tools were identified for developing disinvestment proposals, but generic materials for developing the program theory or rationale and defining the program logic would be useful [156–164], as would business case proformas and communication templates.

Proposals are more likely to be successful if they have certain favourable characteristics and new initiatives are more likely to be sustainable if there is appropriate availability and adequate provision of critical factors to achieve and maintain the proposed components and activities [20, 165–167]. A checklist of the factors influencing likelihood of success and sustainability is available [8].

### 4. Implementation

Some successes with national approaches to disinvestment have been reported and may have elements that are generalisable to local circumstances [72, 85, 102]. However in some circumstances national approaches are not applicable at state/provincial, regional or institutional levels; for example removing or refining indications for reimbursement for ‘low value’ TCPs in national fee-for-service schemes for doctors in private practice may not apply to doctors working in state funded hospitals.

As noted above, there are also many examples in the EBP and quality and safety literature of successful projects at local level to remove unsafe or ineffective TCPs which are not labelled as disinvestment.

Many articles about disinvestment do not address implementation at all and some note that there are difficulties related to implementation but offer no solutions. Of those that do consider implementation, many of the comments are principles, captured in the section above, or barriers and enablers, captured below.

One recommendation for successful implementation is that “*we could create conditions that make it easy for people to avoid using low-value health care services*” [128]. Environmental changes such as closing services, physically removing products from storerooms and work areas, and eliminating items from formularies and purchasing catalogues should achieve this aim and result in complete cessation. In addition, if providers or recipients of a TCP, program or service receive financial reimbursement, removal of funding is likely to reduce use considerably, although not necessarily completely [64, 72, 117, 168, 169]. But not all disinvestment decisions can be managed with structural changes.

The need for an implementation strategy for each disinvestment activity is widely acknowledged. One disinvestment guideline details eight generic steps in their Action Plan [35], the SHARE Program used the SEAchange model for evidence-based change [41] to implement disinvestment pilot projects and support services [9, 10], and a model for ‘de-adoption’ utilises the ‘Knowledge to Action’ framework [22, 170].

A range of approaches to facilitate implementation of disinvestment decisions has been proposed. These include communication and educational materials [58, 72, 78, 117, 121, 171]; financial incentives and pay-for-performance [59, 64, 72, 117, 168]; reinvestment of resources saved [29, 78, 80, 172]; clinical champions [48, 80]; clinical pharmacists to monitor and advise prescribers [68]; quality standards [59, 117]; professional standards, maintenance-of-certification activities and practice audit [117]; prompts through guidelines, protocols, clinical pathways and decision support systems [5, 58–60, 72, 82, 168, 171]; requirements to report variations from mandatory guidelines [59, 72]; monitoring and reporting of outcomes [72, 78, 168]; public reporting of provider performance [59, 117, 168]; training and re-organisation of staffing and equipment [10, 78]; and “*picking low hanging fruit*” before tackling more difficult projects [80]. These proposals have arisen from qualitative work with stakeholders or been derived from an understanding of implementation science; the papers offering these suggestions for implementation do not report application or evaluation of these strategies in the disinvestment context.

Several authors note that implementation is more likely to be successful if decisions are made at the local level, integrated into everyday decision-making and central to local planning [55, 59, 60, 80]. A well-resourced and well-designed formal priority setting entity is reported to improve implementation of decisions [27, 37, 55, 173]. It provides a recognised vehicle to consider information such as new evidence or local performance concerns, one which has transparent processes and appropriate authority for decision-making and action, where local expertise can be built up and local knowledge utilised. It is thought to “*make contentious decisions more palatable and defensible*” [55].

The SHARE Program used the Technology/Clinical Practice Committee (TCPC) as a formal decision-making structure [2]. After piloting several approaches, the Evidence Dissemination Service mentioned above as a method of identification, was finally implemented within a governance model to ensure maximum adherence [11]. Recently-published, high-quality synthesised evidence was identified and publications reporting evidence of harm, lack of effect or findings of a more cost-effective alternative to current practice were prioritised for dissemination. An Evidence Bulletin summarising an individual publication was provided to the TCPC, which then forwarded it to the department head or committee chair responsible for practice in the specific topic area. A response was required to confirm whether current practice was consistent with the evidence, and if not, what measures were being taken to address this or an explanation of why change was not required. When there was evidence of harm, responses to the TCPC were required within 1 month and the responses, or lack thereof, were reported to the Chief Executive the following month. Responses to other Evidence Bulletins were required in three or 6 months. A total of 175 publications were disseminated, 55 of the Evidence Bulletins required responses. Of the 43 responses received during the evaluation period, 32 reported that local practice was consistent with the evidence, six reported that the evidence was not applicable at Monash Health, three noted that local practice was not consistent with the evidence but provided a justifiable reason, and two reported that remedial action was planned to bring local practice into line with the evidence [11].

Although there are some particular challenges to asking people to stop doing things they believe in [1], the general principles of implementation should apply to disinvestment as they do for any practice change. These are summarised in the SEAchange model and the Knowledge to Action framework: engaging all stakeholders, identifying what is already known about practice change in the topic area from the literature and local knowledge, undertaking an analysis of local barriers and enablers, developing an implementation plan including strategies to minimise barriers and build on enablers, piloting and revising as required, and finally implementing in full [41, 170].

### 5. Monitoring, evaluation and reporting

The Schmidt ‘Framework for disinvestment’ notes that both process and outcome evaluations should be undertaken but provides no details [16]. In their framework for evaluation of priority setting processes, Barasa and colleagues propose measures for both procedure aspects and outcomes [39] and a systematic review summarises a range of performance measures to assess use of ‘low value’ TCPs [174]. The ‘Integrative framework for measuring overuse’ lists measurement tools linked to specific project/program goals and discusses advantages and disadvantages of each approach [38].

The SHARE Evaluation Framework and Plan was created for an organisation-wide program of disinvestment in a local health service network [40]. It was developed in consultation with stakeholders and included evaluation domains, audience, scope, evaluation questions, sources of data, methods of collection and analysis, reporting and timelines. A theoretical framework and taxonomy adapted for evaluation and explication of disinvestment projects was also used to understand the process of disinvestment in the SHARE Program [9].

The deficiencies in available economic and usage data and lack of methods for quantifying savings are considered to be significant limitations to evaluation [46, 60, 82, 175, 176].

There are many generic guidance documents for monitoring and evaluation of health programs and projects in a range of settings. Like implementation, the principles, methods and tools for evaluation should be as appropriate for disinvestment as they are for any healthcare improvement project.

Findings from monitoring and evaluation activities should be reported on a regular and/or scheduled basis to the appropriate stakeholders in accordance with project terms of reference, governance protocols and other local requirements.

### 6. Reinvestment

This step will only apply in certain projects when it is anticipated that firstly resources will be released and secondly that they will be available for use elsewhere. Although there is considerable discussion about the potential for reinvestment or reallocation, there is little information about how to do it [1]. Resource release and reallocation are built into prioritisation processes for budget-setting but are not integral to other methods of disinvestment. One proposal for a “*sensible, well-managed reinvestment program”* describes *“a cost-accounting process to capture, and a financial strategy and analysis to return, a pre-agreed portion of real savings*” [172]. However the comments by other authors regarding inconsistencies in accounting practices, insufficient valid and reliable data, lack of methods and tools and absence of reported examples suggest that this may not be currently achievable [1, 21, 48, 60, 83, 175–177].

### 7. Dissemination and diffusion

These terms have been used with specific, but inconsistent, meanings in the disinvestment literature. For example, diffusion has been used to refer to uptake of ‘new’ technologies where disinvestment is used for removal of ‘old’ technologies [178]. In contrast, diffusion and discontinuation have been used to represent ‘spontaneous’ uptake and removal of technologies where dissemination and disinvestment are their counterparts for ‘managed’ uptake and removal [21]. The former links disinvestment with diffusion, the latter with dissemination.

Since the focus of this framework is on implementation of change, and does not differentiate between implementation of investment or disinvestment decisions, the definitions of dissemination and diffusion are taken from the knowledge translation literature (Table 4) [20, 170, 179]. Dissemination involves planned, active processes to share and spread information; diffusion is unplanned and passive.

Outcomes of disinvestment projects should be disseminated to others working in this area to fill gaps in knowledge, avoid duplication, build on successes and learn from mistakes and misfortune. However no guidance for systematic dissemination or facilitation of diffusion of successful disinvestment initiatives at the local health service level was identified. Guidance from the knowledge translation, EBP, QI and implementation science literature for dissemination and diffusion of new TCPs may be a useful starting point, however the specific challenges of disinvestment may influence the generalisability of these methods [1, 180].

### 8. Maintenance

Maintenance is the final step in the change process. It involves strategies to sustain recently implemented change after project support is removed; to integrate the change into organisational systems, processes and practices; and to attain long-term viability of the change (Table 4). Several terms are used in the broader health literature to capture this concept; examples include adoption, assimilation, sustainability and institutionalisation. Sustainability has been used in the context of disinvestment [3, 8, 22, 169, 181]. Maintenance is used in this framework to avoid confusion with use of the term ‘sustainability’ in a different context in the title of the SHARE Program. Maintenance is also used in the evaluation literature to assess *“the extent to which a program becomes institutionalized or part of the routine organizational practices and policies”* and can be applied to both the population targeted for behaviour change and the organisation that enacted or adopted the policy [182].

Montini and Graham propose that the disciplines of *“Science and technology studies, the History and philosophy of science, the Sociology of health and illness, and Medical Anthropology”* be explored to understand the factors relating to sustaining change related to ‘de-implementation’ [169]. Niven and colleagues recommend that ‘de-adoption’ interventions include a sustainability plan to prevent healthcare providers knowingly or unknowingly reverting to old practices [22].

The SHARE Program applied, adapted and developed methods and tools to facilitate sustainability of disinvestment-related initiatives at both the program and project level.

- SHARE projects were assessed against a framework for sustainability based on five categories: structure, skills, resources, commitment and leadership [8].
- The SEAchange model for sustainable, effective, appropriate evidence-based change in health services applied in SHARE projects includes formal assessment of sustainability at each step in the change process [41].
- The determinants of effectiveness outlined in a framework and taxonomy adapted for evaluation and explication of SHARE disinvestment projects could be considered in developing strategies for sustainability of new disinvestment interventions [9].
- The preconditions and underlying principles derived from the literature and local research in development of the SHARE model for exploring sustainability in health care by allocating resources effectively in the local health service setting were identified as factors related to success and sustainability of the whole SHARE Program [8].

## Barriers and enablers

The terms barrier and enabler are commonly used to describe factors influencing the success of change initiatives in health care, but interestingly they are less frequent in the disinvestment literature. Most authors refer to the ‘challenges’ related to disinvestment, few refer to specific ‘barriers’. ‘Enablers’ or existing factors that could facilitate desired change are rarely mentioned, however many authors describe favourable conditions that represent the absence of specific negative factors or strategies that seek to remove them. The challenges and negative factors identified are interpreted as barriers and summarised in Table 10.

**Table 10:** Examples of potential barriers to disinvestment

Common to all aspects of disinvestment ▪ Lack of common terminology, theories, tested frameworks and models, proven methods and tools ▪ The word ‘disinvestment’ generates negativity and mistrust ▪ Divergent understanding of the concept of disinvestment between researchers and health service decision-makers ▪ Lack of guidance and/or successful examples to follow ▪ Lack of resources particularly time, funds and skills ▪ Lack of any of the elements of the framework ▪ Resistance to change
Establishment and delivery of program ▪ Lack of communication between agencies ▪ Autonomy of agencies resulting in multiple different systems ▪ Wastage of resources by duplication of effort, particularly in HTA ▪ Lack of resources to support policy mechanisms ▪ Lack of appropriate data collection systems ▪ Cost of appropriate data collection systems ▪ Lack of political, clinical, or administrative will to achieve change ▪ Difficulty establishing systems and processes to assess choices and reallocate resources across and between programs. Easier when done within programs but this has limited effectiveness. ▪ Difficulty establishing systems and processes between competing sectors or paradigms eg cure versus prevention, acute versus community care, drug therapy versus counselling ▪ Lack of coordination and integration of systems and processes ▪ Short-termism in government policy ▪ Conflicting priorities – at individual levels, and/or between levels ▪ System inertia ▪ Longstanding structures, institutional practices and organisational relationships ▪ Poor understanding of organisational practices and relationships ▪ Lack of established triggers to initiate disinvestment discussions ▪ Scarcity of strategic plans that include disinvestment ▪ Lack of incentives, presence of disincentives ▪ Fee for service models reward quantity not quality
Stakeholder engagement ▪ Lack of stakeholder commitment ▪ Stakeholder inertia ▪ Difficulty identifying and engaging multiple diverse stakeholders ▪ Resistance to, or lack of understanding of consumer participation
Identification of disinvestment opportunities ▪ Health Technology Reassessment (HTR) not conducted routinely ▪ Public and private funding focused on HTA rather than HTR ▪ Insufficient ‘unequivocal’ evidence to disinvest ▪ Lack of mechanisms to identify disinvestment targets ▪ Difficulties in producing, accessing & interpreting economic data ▪ Willingness to use lower quality evidence to maintain status quo
Prioritisation and decision-making ▪ Lack of knowledge of available tools ▪ Unfamiliarity with economic evaluations ▪ Disagreement with assumptions in economic evaluations ▪ Difficulties estimating marginal costs ▪ Reluctance to disinvest if there are sunk costs in existing technology and supporting capital infrastructure ▪ Reluctance to expend effort in disinvestment if benefits not clear ▪ Gains from disinvestment are less readily measured and may not happen but losses from disinvestment are immediate ▪ Strength of vested interests and lobby groups ▪ Lack of negotiating skills making it difficult to resist opposition ▪ Conflicting priorities between decision-makers ▪ Conflicting priorities between local, regional and national levels ▪ Reluctance to disinvest due to heterogeneity of outcomes and/or if there is potential for benefit in some subgroups or individuals ▪ Controversy associated with removal of an effective TCP in favour of a more cost-effective alternative and/or where there is lack of evidence of effect but general perception that it works ▪ Sensitivity of disinvestment target eg children, cancer, end of life ▪ Lack of decision-making processes ▪ Lack of integration with other decision-making processes ▪ Requirement for prospective data collection or further research to provide enough information for decision ▪ Difficulty making choices and reallocating resources across and between programs. Easier when done within programs but this has limited effectiveness. ▪ Difficulty making choices between competing sectors or paradigms eg cure versus prevention, acute versus community care, drug therapy versus counselling ▪ Decision-makers not held in sufficiently high regard for decisions to be respected and enforced ▪ Perceived influence of power imbalances and hidden agendas ▪ Political challenges
Implementation ▪ Inadequate project timelines ▪ Lack of funding for implementation ▪ Lack of skills in project management ▪ Lack of skills in change management ▪ Loss of patient choice ▪ Loss of perceived entitlement to treatment ▪ Loss of clinical autonomy ▪ Clinician reluctance to remove practices they perceive as integral to their professional practice and identity ▪ Loss of perceived benefit of intervention being removed ▪ Perceived criticism of practice and/or practitioners ▪ Perception that management priority is only to save money ▪ Lack of incentives, presence of disincentives ▪ Lack of data to substantiate need ▪ Gains from disinvestment less readily measured and may not happen, but losses from disinvestment are immediate ▪ Complexity of practice change if disinvestment limited to certain groups or for certain indications ▪ Lack of coordination between projects resulting in gaps and duplication ▪ Stakeholder fatigue and disillusionment with constant change
Monitoring and evaluation ▪ Routinely-collected data not valid or reliable, often out-of-date ▪ Routinely-collected data not precise or specific enough ▪ Cost of obtaining appropriate data ▪ Lack of post-market surveillance ▪ Lack of methods to quantify savings ▪ Distrust of reasons for monitoring and evaluation
Reinvestment ▪ Lack of methods for reallocating resources released ▪ Lack of examples of successful reinvestment ▪ Some cost savings may not be realised eg length of stay reduced but beds immediately filled with other patients of greater acuity
Research ▪ Assumptions that current practice is effective ▪ Ethical objections to randomising patients to control groups ▪ Resistance to enrolling patients in trials due to belief in intervention ▪ Difficulty getting funding to research existing practices

Some barriers impact on all aspects of disinvestment across each level of influence [15, 16, 21, 24, 29, 48, 58, 78–80, 83, 116, 120, 129, 175, 178, 183–187]. Barriers to establishment and delivery of a program for decision-making are noted [8, 9, 24, 31, 55, 58, 64, 79, 82, 120, 131, 132, 153, 175, 183] and other barriers are categorised using the steps of the disinvestment process: stakeholder engagement [2, 58, 78–80, 82, 120, 153], identification of disinvestment targets [8, 9, 16, 21, 24, 46, 48, 58, 60, 63, 72, 79, 82, 120, 129, 175, 183, 188–190], prioritisation and decision-making [2, 21, 24, 31, 46, 48, 55, 58, 60, 63, 64, 72, 79, 82, 120, 129, 132, 175, 183, 188, 190, 191], implementation [2, 8, 21, 46, 58, 64, 79, 82, 120, 132, 153, 169], monitoring and evaluation [8, 46, 48, 60, 82, 175], reinvestment [55, 64, 153, 175, 176] and research [58, 183, 189]. There is some overlap where the same barriers affect more than one aspect of the process.

This summary only captures barriers to disinvestment activities. Barriers and enablers to investment in new TCPs and strategies to address them are summarised elsewhere [2]. Programs for disinvestment may require system reform, so the barriers inherent in large-scale change will also be applicable. The body of literature on barriers and enablers to using evidence in decision-making and implementing practice change will also be relevant to disinvestment activities.

In addition to the list summarised here and the wider literature, an analysis of local barriers and enablers should be undertaken for every project to identify crucial contextual factors.

## Discussion

### Limitations

Although a rigorous systematic approach was taken, it is impossible to be comprehensive in ascertaining all the relevant literature on disinvestment; the reasons are outlined in the conceptual review [1]. As a result, some relevant publications may not have been identified and some information may not have been published. Despite these limitations, the messages about operationalising disinvestment are generally clear and consistent and provide strong underpinnings for the framework.

The literature has been reviewed from the perspective of a local health service, however the authors’ experience is based in the Australian health system; hence differences with other health systems may not have been recognised and additional decision-making settings or methods and tools may have been missed.

The specific details of the ‘where, who and how’ of decision-making is likely to differ between organisations but the underlying premises should be the same: individuals and groups make decisions under certain conditions. The classifications of decisions and decision-makers might be useful starting points to elucidate local particulars.

The proposed framework is conceptual and untested. Naming of categories, determination of their constituent elements and the relationships between components has not been piloted or refined with stakeholder input. It is large, complex and all-encompassing and may prove too daunting or complicated to be achieved in this format. Testing and research may establish if it is feasible in the current overarching format or if it should be renamed, redefined or reformulated for implementation in smaller sections.

The framework is proposed at the ‘big picture’ level and requires supplementation with detail for all the components. There are some existing frameworks, models, methods and tools that can be applied in several areas but not for all elements within the framework.

There are many barriers that cannot be addressed by generic system changes and must be tackled when implementing the framework in individual situations. Many of these may be successfully overcome with local strategies; however some aspects of the framework involve potentially insurmountable barriers in the current environment. The main example is lack of valid, reliable, timely, appropriate and sufficiently specific data to identify disinvestment targets and monitor and evaluate disinvestment initiatives.

### Implications for policy and practice

As the focus of this review is operationalisation of disinvestment, the implications for policy and practice have been integrated throughout the paper.

### Implications for research

The implications for research in operationalising disinvestment are enormous. Placing the research component of the proposed framework across all the constituent elements illustrates that there is a need for research in each of them. Some topics stand out as priorities.

Many authors report a lack of frameworks, models, methods or tools for disinvestment. However there are some frameworks and models for disinvestment, although not tested; and plenty of methods and tools, many of which are tested, frequently from other research disciplines but which are relevant for disinvestment projects. Perhaps a more important factor is the lack of proactive mechanisms, prompts and triggers [9, 11, 16, 21, 24, 27, 192]. There are rigorous methods for HTA and analysis of health service data but no systematic methods to initiate these processes or draw the results to the attention of health service decision-makers. It is also not clear who is, or should be, responsible for instigating and making decisions and taking action. Research in these areas is a priority.

Investigation of data requirements, data collection methods and skills of decision-makers to use data for disinvestment is another priority [21, 24, 27, 46, 48, 55, 58, 60, 131]. Support for data collection after a TCP has been introduced is low and research into methods and resources required for post-market surveillance and “*coverage with evidence development*” is required [24, 132].

Some authors have highlighted other aspects of disinvestment for research such as exploring disinvestment at local health service and individual practitioner level [16, 55, 56, 80, 188, 193], taking a longitudinal approach from inception through implementation to outcomes that cross organisational boundaries [80, 188], identifying determinants for disinvestment [15, 80, 129], implementing change management [56, 58], and drafting and refining frameworks, methods and tools [15, 24, 29, 58, 129, 175, 184, 185]. Mechanisms to develop, implement and evaluate disinvestment activities can be built on existing theoretical frameworks from other research paradigms such as HTA, knowledge translation and implementation science [28, 83]. Measures of impact, potential unintended consequences and factors contributing to success or failure also need to be captured [24, 83, 193]. The SHARE Program provides some early work to build on by reporting disinvestment projects from inception to implementation [9]; identifying determinants for disinvestment, potential unintended consequences and factors contributing to success or failure [9]; and developing frameworks, models and algorithms [5–9, 11] and evaluation frameworks and plans [10, 11, 40]. These outputs of the SHARE Program are summarised in Paper 1 [3].

Research could also include testing the proposed framework in different contexts.

## Conclusions

There is no agreed definition or common understanding of disinvestment, yet the concept is widely discussed and disinvestment initiatives and research are called for. Although there are only a few, largely untested, frameworks and models and little practical guidance in the literature, there are clear and consistent messages regarding principles for decision-making, settings and opportunities to identify disinvestment targets, steps in the disinvestment process, methods and tools, and barriers and enablers. This information has been drawn together into a framework for operationalising disinvestment in a systematic, integrated, organisation-wide approach in the local healthcare setting.

Definitions for essential terms are proposed and key concepts underpinning the framework have been made explicit. The term disinvestment is used in the broadest sense, ‘removal, reduction or restriction of any aspect of the health system for any reason’, and can be applied to products, devices and equipment; clinical practices and procedures; health services and programs; information technology and corporate systems. Given the negative connotations of the word and the problems inherent in considering disinvestment in isolation, the basis for the framework is ‘resource allocation’ addressing the spectrum of decision-making from investment to disinvestment.

The framework is based on three components: the program consists of principles for decision-making and settings that provide opportunities to introduce systematic prompts and triggers to initiate consideration of disinvestment; projects follow the steps of the disinvestment process; and research is needed across all aspects of the framework.

The proposed framework can be employed at network, institutional, departmental, ward or committee level. It is proposed as an organisation-wide application, embedded within existing systems and processes, which can be responsive to needs and priorities at the level of implementation. It can be used in policy, management or clinical contexts, for resource allocation and potentially other decision-making processes.

There are many theories, frameworks, models, methods and tools from other areas of health research and practice that are relevant to disinvestment which could be employed within this framework.

Multiple barriers to establishing a decision-making framework and implementing disinvestment initiatives were identified. Some of these relate to the lack of elements that form individual principles and are addressed in the framework, however many involve local factors that can only be tackled when implementing the framework in particular contexts.

The framework captures all the identified information from the literature about operationalisation of disinvestment in the context of resource allocation. This could be a strength, if all the elements are required for a robust effective program of decision-making and action, or a weakness, if it is too complex to be achieved in practice.

## Additional file

Additional file 1: Principles for resource allocation. (PDF 697 kb)

## Abbreviations

A4R: Accountability for Reasonableness
EBP: Evidence Based Practice
EVIDEM: Evidence and Value: Impact on DEcision Making
HsW: Health Sector Wide
HTA: Health Technology Assessment
HTR: Health Technology Reassessment
MCDA: Multi-criteria decision analysis
NICE: National Institute of Health and Clinical Excellence
PBMA: Program Budgeting and Marginal Analysis
QI: Quality Improvement
SHARE: Sustainability in Health care by Allocating Resources Effectively
STEPPP: Systematic Tool for Evaluating Pharmaceutical Products for Public Funding Decisions
TCPC: Technology/Clinical Practice Committee
TCPs: Technologies and Clinical Practices
