# Supplementary material for: Sustainability in Health care by Allocating Resources Effectively (SHARE) 10: operationalising disinvestment in a conceptual framework for resource allocation
Source: BMC Health Serv Res. 2017 Sep 8;17:632. doi: 10.1186/s12913-017-2506-7 (PMC5590199; doi:10.1186/s12913-017-2506-7)
Supplement: Additional file 1: — Principles for resource allocation. (PDF 697 kb) [file 12913_2017_2506_MOESM1_ESM.pdf]

Sustainability in Health care by Allocating Resources Effectively (SHARE) 10: Operationalising disinvestment in a conceptual framework for resource allocation

Additional File: Principles for resource allocation

Contents

Categories 2
Descriptions 4
Relationships 7
Methods and Tools 8
References 10

Categories

Forty-two principles were identified from the existing literature and the SHARE publications and grouped into eight categories to add further meaning and context. These are Boundaries, Ethics, Governance, Structures, Processes, Stakeholder involvement, Resources and Preconditions.

Boundaries

Clear boundaries should be established to define the parameters that the framework will operate within. At a minimum, this should include context, scope and timeframe; with additional factors where relevant. Context can play a significant role in decision-making for disinvestment or resource allocation and should not be underestimated [1-3]. Explicit statements of context and scope enable identification of all relevant stakeholders [4]. It is proposed that frameworks are implemented either as long-term ongoing programs or within a defined timeframe, such as five years, and adequate resources should be provided to achieve this [2, 5-12]. There is also a need for clear timelines around implementation of policies and delivery of outcomes [7, 8].

Ethics

Ethical frameworks for decision-making in situations of limited health resources have been produced by governments, health agencies and research bodies [13-19]. Most elements are common to all, and are also cited frequently in the literature as necessary in the disinvestment process. These are justice, fairness, equity, access, legality, honesty, clinical ethics, patient autonomy and privacy.

The terms justice, fairness and equity are frequently used together as if to convey that they have different meanings, but they are also used interchangeably. There are a range of definitions for these terms, with fairness and equity often used to define justice. Although there is considerable overlap in definitions, the frequent use of all three together suggests that authors wish to convey subtle differences. To reflect these sentiments all three have been included and definitions developed to differentiate between them in the context of this framework.

There are many types of justice. Distributive justice is used to consider what is right or just in the allocation of goods within society [20]. Distributive justice and social justice are both used to depict the concepts of fairness and equity [16, 21]. Procedural justice relates to decision-making which is included below in Processes [22]. The other forms of justice, mainly related to the legal system, are not relevant to this context. For the purposes of this framework, the principle considered here is utilitarian justice, maximising outcomes through application of resources for the greatest benefit for the most people.

Two approaches to equity in health care have been described: equity related to a concept of need and equity related to access to services [23]. For clarity, equity is being used here in relation to need and access to services has been included separately.

The four components of the Accountability for Reasonableness approach to decision-making are frequently referred to in the disinvestment literature as "*ethical factors*": 1) the process must be public and fully transparent, 2) decisions are based on reasons that stakeholders agree are relevant, 3) decisions can be revised on appeal and 4) there should be assurance through enforcement that these conditions will be met [24]. These principles are not addressed here under Ethics but are integrated into other sections of the framework: transparency and enforcement in Governance and relevance of decision-making criteria and appeals in Processes.

Governance

The principles of governance are transparency, accountability, authority, enforcement, sound management and quality improvement.

Authors note that transparency, accountability and enforcement enable fairness and equity; sound management ensures that programs and projects are delivered effectively and efficiently; and quality improvement encourages learning and ongoing development.

Structures

The desired elements of structures for decision-making in resource allocation include a systematic approach, integration, alignment, monitoring and evaluation and reporting. A systematic, integrated, aligned approach is seen to enable transparency and accountability [8, 25] which in turn enables fairness and equity [26].

It is anticipated that integration of decision-making systems and processes into existing infrastructure, alignment with local priorities and strategic objectives, and embedding the operational aspects within business plans and routine planning activities will increase the likelihood of success and sustainability and normalise the concept of disinvestment as part of day-to-day decision-making [5, 8, 9, 12, 27-30].

Integration should be system-wide at the level in which the framework is being implemented eg network, institution, department, ward, committee [5, 8, 31, 32]. This will allow all opportunities for disinvestment to be included [6, 33, 34]; shared decision-making with all stakeholders across the relevant health economy [7, 31, 35-37]; consideration of the impact of decisions on other systems, organisations and departments [5, 7, 14, 36, 38, 39]; consultation between policy-makers, business managers, clinicians and consumers [7, 39-41]; institutional learning leading to improvement [42]; and collaboration with teams working in related areas such as outcomes research, quality improvement, patient safety and system redesign [32, 37].

Processes

Processes for decision-making about resource allocation should be robust [2, 4, 7, 8, 11, 25, 28, 34, 43] and many authors cite the Accountability for Reasonableness approach as a way of achieving this [6, 16, 38, 43-46].

A robust process is based on explicit criteria, is informed by evidence, includes analysis of risks and benefits, is internally and externally consistent, has mechanisms to revise or appeal decisions, and includes effective communication activities.

There is a huge range of potentially relevant criteria for resource allocation decisions. Most authors emphasise that a list of criteria should be developed with input from all stakeholders to meet the objectives of individual situations. The commonly cited basic requirements include clinical parameters such as safety and effectiveness, economic measures such as cost-effectiveness and affordability, and social factors such as local values and priorities. Additional criteria will depend on the setting and context.

Stakeholder involvement

It is universally acknowledged that all good decision-making requires stakeholder engagement and virtually all authors writing about disinvestment, resource allocation or priority setting refer to this fundamental issue. Stakeholder empowerment refers to the ability of stakeholders to contribute to and influence decisions [47].

Although there is extensive literature on the effects of patient involvement in decisions about their clinical care [48-50], there is no clear evidence about the impact of patient or public participation in collective decisions for healthcare policy and service delivery [51-54]. However there is a growing body of work investigating methods for engaging and empowering a range of stakeholders in this generic context [8, 47, 51, 55, 56] and, more specifically, in resource allocation [52, 57, 58] and disinvestment [59-62].

Resources

The proposed activities require adequate and appropriate resources to be effective and sustainable [12, 31, 36, 37, 40, 63-68]. These include funding; time; access to high quality analytics of information such as research evidence, population health data, local health service utilisation data and economic analyses; expertise; methods and tools. Several authors call for dedicated resources and in-house "*resource centres*" to provide expertise; access to relevant methods and tools; and education, training and capacity-building [8, 9, 65, 69-72].

Preconditions

Certain preconditions must be fulfilled before program and project objectives can be achieved. Strong leadership and commitment is required at every level, as is influence and support. The organisation must be ready to change and the internal and external environments must be favourable.

Descriptions

**BOUNDARIES**	
**Context**	Specify the context where decisions will apply. These might include, but are not restricted to, 1) acute, subacute, rehabilitation, community or mental health services; health promotion and education programs; or residential aged care at 2) region, local network, institution, department, ward or committee. [1-3]
**Scope**	Specify the type of decisions and topics to be addressed. These might include, but are not restricted to, policy, management or clinical decisions to address capital works, plant and equipment; human resources; organisational systems and processes; guidelines and protocols; procurement or commissioning of TCPs, models of care or health programs and services. [4, 73]
**Timeframes**	Specify timeframes for decision-making programs (eg long-term ongoing or defined limited application such as 5 years), implementation of decisions and delivery of outcomes. [2, 5-12]
**ETHICS**	
**Justice**	Maximise outcomes; direct resources for the greatest utility or benefit for the most people, the 'greatest good for the greatest number'. [13, 15, 16, 18, 20, 22, 74, 75]
**Fairness**	Act impartially; not discriminating on the basis of race, nationality, colour, language, religion, gender, marital status, sexual orientation, social status, political or other opinion, capacity to pay, location of residence, ownership of property, the need for treatment arising out of past behaviour, or age (except where age may affect the outcome). [6, 13, 14, 16-18, 26, 39, 43, 66, 75-78]
**Equity**	Horizontal equity: Offer treatment to all patients that meet the relevant criteria, or to none; 'treating like cases alike' or 'equal access for equal clinical need.' The decision should be made for all patients in a group with similar clinical need and not for individuals. Vertical equity: Provide unequal but equitable treatment for people with unequal health needs by giving priority to groups with greater need, for example disadvantage due to social determinants of health. [2, 13-18, 21, 23, 26, 31, 38-40, 47, 78, 79]
**Access**	Ensure consumers or communities are able to use appropriate services determined by five dimensions of accessibility (approachability, acceptability, availability and accommodation, affordability, appropriateness) and five abilities of populations (ability to perceive, seek, reach, pay and engage). [14, 16, 23, 40, 43, 66, 78, 80]
**Legality**	Act within the law. Ensure decisions are made by those who are legally accountable for the resources and not made by external groups such as pharmaceutical companies, research bodies, or others with vested interests. [7, 14, 39]
**Honesty**	Be truthful. Do not lie or hide things. [7, 78]
**Clinical obligations**	Guarantee that removal, reduction or replacement of services or TCPs do not compromise clinical ethical obligations, such as beneficence, or other professional standards. [74]
**Patient autonomy**	Empower and encourage patients to make informed decisions about their treatment. Safeguard patient choice and informed consent. [16, 59, 74]
**Privacy**	Ensure patient confidentiality at all times. [74]
**GOVERNANCE**	
**Transparency**	Make all elements clear and visible eg who makes decisions, how decisions are made, reasons for decisions, how they are documented, how they will be implemented and evaluated. Seek declarations of conflict of interest and address them openly. Implement single system ie no parallel system where those who lobby could get undue priority. Record departures from process and subject them to scrutiny. [2, 6-8, 11, 12, 14, 15, 17, 25, 26, 29, 31, 32, 36, 38, 42, 43, 46, 47, 59, 62, 63, 65, 66, 75, 76, 78, 81-83]
**Accountability**	Ensure decisions are only made by those who have the authority to do so. Make the lines of authority and responsibility clear and be prepared to acknowledge if errors or complications occur and be accountable for correcting them. [2, 6, 15, 27, 29, 36, 39, 43, 62]
**Authority**	Ensure decision-makers have the knowledge and capability to make the decisions, the control and power to enact them, and the ability to move resources within and between programs, services, facilities, etc as appropriate. [6, 27, 40, 75]
**Enforcement**	Implement mechanisms to ensure firstly that all principles are adhered to and secondly that decisions are enacted as planned. [6, 16, 18, 43-47, 75, 84]
**Sound management**	Establish sound organisational, performance management and resource management structures to ensure due process is followed and implementation of decisions is achieved. Include appropriate corporate expertise from areas such as Finance, Human Resources, Contracting, Communications, Procurement, etc. [5-9, 31, 39, 40, 83]
**Quality improvement**	Embed opportunities for ongoing reflection on the processes and outcomes of administration of the framework and take the appropriate actions to increase effectiveness, satisfaction and other measures relevant to the stated objectives. [40, 42, 85]

STRUCTURE	
**Systematic approach**	Establish systems that are planned, methodical, purposeful and coherent and do not rely on ad hoc, impromptu or improvised mechanisms for decision-making and change. [2, 3, 5, 6, 9, 25, 37, 41, 43, 83, 86]
**Integration**	Incorporate decision-making systems and processes for resource allocation into existing infrastructure and implement system-wide at each level ie region, local network, institution, department, ward or committee. [2, 5, 6, 9, 11, 14, 27-29, 32, 34, 35, 39, 42]
**Alignment**	Align decision-making systems and processes with the institutional mandate, priorities, strategic goals and objectives. Integrate operational aspects within relevant business plans. [2, 5, 9, 11, 12, 29, 31, 37, 68]
**Monitoring and Evaluation**	Assess compliance with, and effectiveness of, the administration of the program to enable improvement in the systems and processes. Assess outcomes of decisions introducing, removing, reducing or replacing services or TCPs to inform ongoing use and appropriateness of funding. [11, 13, 31, 38, 40, 42, 43, 47, 85]
**Reporting**	Report outcomes of monitoring and evaluation to relevant stakeholders in a transparent and timely manner to enable enforcement and quality improvement and inform future decisions. [7, 13, 29, 40, 87]
**PROCESS**	
**Explicit criteria**	Develop appropriate and achievable criteria to meet the desired objectives, document them explicitly and adhere to them in the decision-making process. [2, 6, 8, 11, 15, 16, 18, 26, 29, 39, 40, 43, 75, 86]
**Evidence-informed**	Use the best available evidence for each of the specified criteria. This may include published research or research syntheses (eg systematic reviews, health technology assessments and evidence-based guidelines), population health data, health service utilisation data, cost data, health economic analyses or models, consumer and staff perceptions, or other sources. [1, 5, 7-9, 12, 16, 20, 25, 26, 29, 31, 32, 34, 37-39, 43, 47, 64, 65, 67, 68, 75, 78, 86, 88-92]
**Risk-benefit analysis**	Assess the risks and benefits of introducing, continuing, expanding, removing, reducing, restricting or replacing individual services or TCPs. Assess the risks and benefits of implementing a significant change initiative. [14, 15, 37, 43, 78, 87]
**Consistency**	Internal consistency: Ensure that the systems, processes, values and reasoning that underpin the program are consistent. In some cases, standardisation may be beneficial.<br>External consistency: Ensure that local programs are consistent with regional programs, regional programs are consistent with national programs, etc.<br>Consistency of information: Ensure that all materials used in communication are consistent with each other and with the systems, processes, values and reasoning of the program.<br>[14, 15, 18, 26, 39, 42, 43, 66, 78, 82, 93]
**Appeals process**	Establish formal mechanisms, transparent rules and requirements, to review, revise or appeal decisions. Correct errors and address disagreements constructively. [13, 15, 18, 38, 39, 42, 47, 78]
**Communication**	Document decisions.<br>Develop channels of communication, methods and tools to:<br>▪ Convey information to stakeholders so they are aware of processes, requirements, decisions and actions taken.<br>▪ Seek input from stakeholders to identify issues and drive decisions.<br>▪ Seek feedback from stakeholders to evaluate the processes and outcomes of making and implementing decisions.<br>▪ Ensure ‘top down’ and ‘bottom up’ mechanisms to convey information and seek input and feedback are available, promoted to stakeholders and user-friendly.<br>▪ Distribute information to mass media and social media to educate and inform the community and facilitate public dialogue on healthcare decisions.<br>▪ Share information with the international community to avoid duplication of effort by publishing assessments, decisions, project initiatives and research activities.<br>[7, 11-13, 15, 31, 32, 37, 42, 57, 66, 87]
**STAKEHOLDER INVOLVEMENT**	
**Engagement**	Identify all relevant stakeholder groups, internal and external to the program. Examples include, but are not restricted to, government departments, local authorities, health agencies, health services, professional associations, representative organisations, advocacy groups, policy makers, managers, health practitioners, researchers, resource personnel (eg systematic reviewers, data analysts, health economists, etc) and representatives of the public. Public participation can involve patients, service users, consumers, community members, citizens, taxpayers, voters, etc. Select an appropriate model, framework or guidance document to follow and use methods and tools for stakeholder engagement relevant to the setting and context.

**Empowerment**	Ensure that stakeholders have the power to contribute to and influence decisions. Implement mechanisms to minimize the effect of the power differences among actors in healthcare organizations; for example give each stakeholder equal opportunities to participate at different stages of the decision-making [47].
**RESOURCES**	
**Funding**	Provide adequate funding to underpin the systems and processes to make, implement and evaluate decisions. [11, 12, 31, 36-38, 43, 68, 94]
**Time**	Allow all relevant stakeholders to take sufficient time for participation. [1, 6, 7, 27, 52, 63, 95]
**Expertise**	Ensure appropriate expertise is available to make, implement and evaluate decisions. Relevant expertise includes, but is not restricted to, finding and using information, health technology assessment, health economics, data analysis and interpretation, negotiation and meeting facilitation, project management, change management, health program evaluation and knowledge and experience in the topic under consideration. [1, 2, 5, 8, 9, 11, 12, 27, 32, 37, 43, 67-70, 96-98]
**Information**	Provide adequate and appropriate access to high quality information to underpin decisions including, but not restricted to, research evidence, population health data, local health service data, consumer feedback and economic analyses. [2, 6, 7, 27, 31, 38, 40, 42, 43, 98]
**Methods and tools**	Assist decision-makers, implementers, evaluators and support personnel to find and use appropriate, valid and reliable methods and tools relevant to program and project activities. [1, 2, 5, 8, 9, 11, 27, 32, 43, 67, 69, 70, 96-98]
**PRECONDITIONS**	
**Leadership**	Appoint and train established and emerging leaders with strengths in negotiation and conciliation, political and cultural awareness and sensitivity. [2, 5-8, 12, 27, 31, 32, 37, 41, 42, 68, 87, 97, 99]
**Commitment**	Establish the program in a way that allows those who are responsible and accountable, the leaders and champions, the decision-makers and support staff to be fully and openly committed, dedicated and loyal to the principles and practices within it. [2, 5-8, 27, 31, 32, 41, 42, 87, 97, 99]
**Influence**	Engage key stakeholders with sufficient and appropriate influence in relevant areas to facilitate and enable rigorous decision-making and effective action. Considerations might include, but are not restricted to, level of seniority, authority, credibility amongst peers, representation on relevant committees, extent of internal and external networks, etc. [5, 12, 37, 40, 47, 52, 68, 100-102]
**Support**	Provide support to those involved by endorsing and promoting decisions, trouble-shooting and problem solving, addressing personal and professional needs, etc. [3, 5, 7, 12, 20, 27, 31, 32, 37, 40, 44, 51, 59, 61, 62, 66, 69, 87, 103-106]
**Readiness for change**	Assess readiness for change at all the relevant levels prior to establishing the program and prior to implementing the decisions taken. Use a valid and reliable instrument. [5, 22, 27, 31, 68, 87]
**Favourable environment**	Consider factors within the internal and external environments that may influence the establishment, delivery and outcomes of the program and what the impacts might be. Examples include, but are not restricted to, setting and context, politics, economic climate, power dynamics and other relationships, priorities, values and culture. [3, 8, 23, 31, 39, 42, 43]
**RESEARCH**	
Consider the role of and opportunities for research in new systems and processes; theories, frameworks and models; methods and tools.	

Relationships

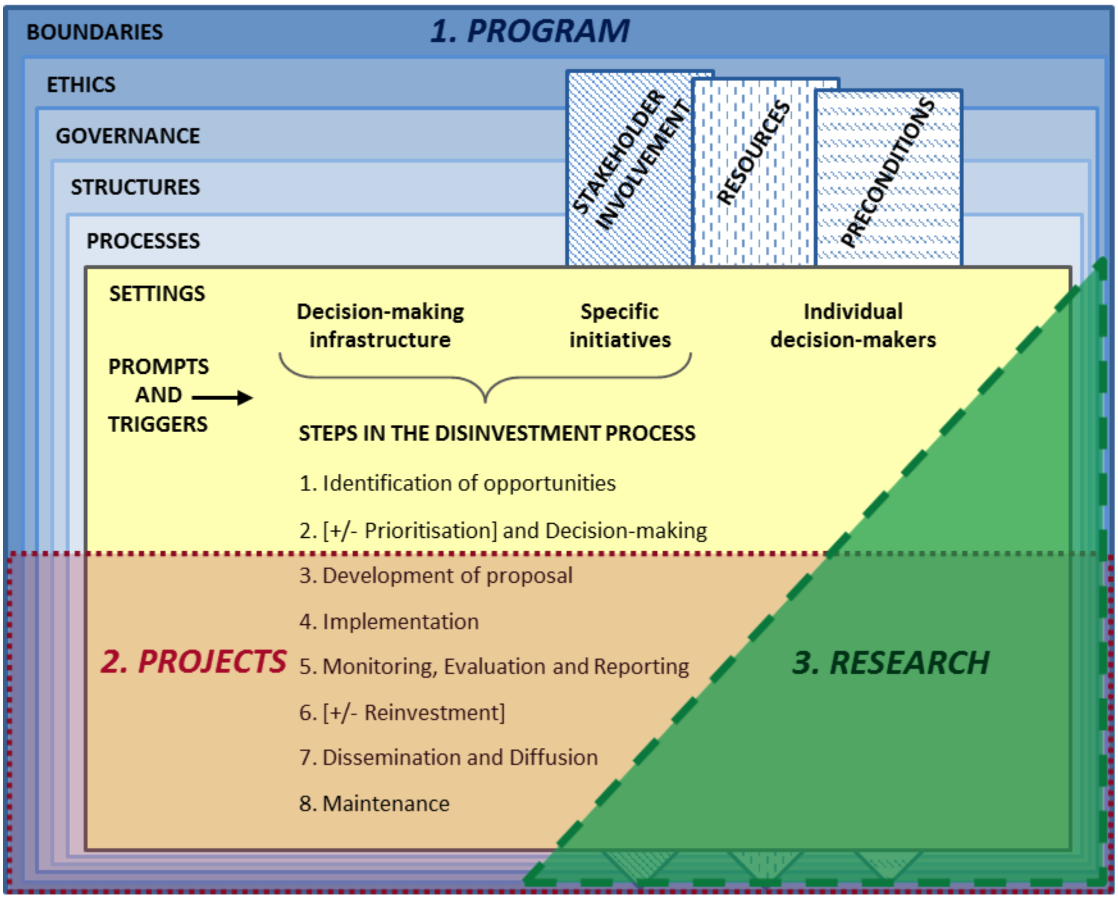

The principles are presented in the framework as two groups.

The first group have a hierarchical relationship depicted as a series of nested boxes. The whole program is defined by explicit boundaries, ethical principles underpin good governance, governance directs and controls structure, and structure enables and accommodates process. The decision-making settings, prompts and triggers all sit within the scaffold of these five categories.

The second group, represented as three vertical bars, are required across all of the other elements. For example, stakeholders need to be involved in defining the boundaries and establishing the ethical parameters and methods of governance; they should be included in the structures and processes and participate in the projects and research. Adequate and appropriate resources and the noted preconditions will be required to establish, maintain and improve all aspects of the framework.

The intersection of the two groups of principles also demonstrates that ethics, governance, structures and processes also apply to stakeholder engagement, resources and preconditions. For example, stakeholder engagement should be systematic and integrated, funding should be sourced ethically and influence should be transparent.

These principles and their relationships also apply to the project and research components.

Methods and Tools

The principles were derived from the disinvestment and resource allocation literature, however they are applicable in most decision-making contexts. Methods and tools to assist in implementation of many of the principles can be found in the wider health or organisational literature, for example instruments to assess leadership or readiness for change and templates for communication strategies.

Two publications provide advice in a range of areas relevant to disinvestment. A book on rationing, priority setting and resource allocation in health care discusses multiple generic and specific methods and tools suitable for disinvestment including stakeholder participation, leadership, economic evaluation and several of the steps in the disinvestment process [114]. A toolkit for decommissioning and disinvestment, defined as withdrawal of funding from the provider organisation, provides high-level guidance on governance and administrative matters for removal of health services, not individual TCPs, and some tools for assessing service performance against UK data [93].

Stakeholder involvement

There are multiple evidence-based handbooks, toolkits and similar documents regarding consumer and community involvement in healthcare decision-making. These include publications produced by international [107, 108], national [109], regional [110], and local agencies [111] as well as discipline/condition-specific publications [112]. In addition to guidance on consumer involvement, resources for engagement of multiple stakeholder groups are also available [99, 113-115].

Guidance more specifically related to topics associated with this overview include involving consumers and/or community members in health policy decisions [116, 117], the HTA process [58, 105] including HTA at the local level [56], decisions about use of health technologies [99, 118, 119], and priority setting [47]. The SHARE Program developed a model for consumer engagement in resource allocation at the local level [57] and an extensive list of all potential stakeholders to consider in decommissioning of local health services has been produced [7].

Resources

Lack of knowledge and skills in evidence-based decision-making, project management, implementation and evaluation and lack of time to carry out the related activities are widely recognised as barriers to effective change in health care generally and resource allocation in particular [1, 2, 8, 9, 27, 32, 36, 43, 67, 69-71, 96-98, 120, 121]. Generic guidance in these areas can be found in the national and international resources noted above and the wider health and organisational literature, however no specific advice regarding provision of resources to address these issues was identified in the disinvestment and resource allocation literature.

The SHARE Program investigated four in-house services to provide expertise and support to decision-makers and project teams: an Evidence Dissemination Service and Data Service to facilitate proactive use of evidence from research and local data; a Capacity Building Service to provide training in evidence-based change, implementation and evaluation methods; and a Project Support Service to provide methodological advice and practical support in project conduct and delivery [71, 121]. The Evidence Dissemination Service was successful in ensuring local practice was consistent with current evidence but was suspended due to the intensive resources required to ascertain, process and disseminate the information. The Capacity Building and Project Support Services were successful in achieving short term objectives, but long term outcomes were not evaluated. The Data Service was not implemented at all due to local factors beyond the scope of the SHARE Program. Local needs analyses, evaluation frameworks and plans, and discussion of factors that influenced decisions, processes and outcomes of the pilot projects may inform others wishing to undertake similar initiatives [71, 121].

Preconditions

In addition to the formal prompts and triggers that can be built into decision-making infrastructure, there are also informal yet systematic approaches that could be integrated into other systems and processes [122]. These are associated with the principles denoted as preconditions. For example, although these strategies may not always identify opportunities for disinvestment themselves, they may create a favourable environment for consideration of disinvestment and readiness to implement change within the organisation. Identifying clinical champions interested in disinvestment and staff who have previously undertaken disinvestment projects and assisting them in future activities facilitates leadership and provides support. Commitment and influence will be evident if discussions about disinvestment are introduced into ‘Leadership Walkrounds’.

Additional systematic methods to facilitate identification of disinvestment opportunities in a local health service

Discuss principles of disinvestment and examples of successful projects at department/unit meetings, educational events, etc
Assign a group member to look for disinvestment opportunities in committee/working party decisions
Add a disinvestment question to the ‘Leadership Walkround’ protocol
Identify clinical champions interested in disinvestment in each program/department/unit who would look out for opportunities
Support staff who have undertaken a disinvestment project to look for more opportunities
Have disinvestment as a high priority in medication safety reviews
Encourage or require projects that are introducing something new to have a component of disinvestment
Review projects that are being conducted for other reasons and identify and focus on any disinvestment elements

References

1. Polisena J, Clifford T, Elshaug AG, Mitton C, Russell E, Skidmore B. Case studies that illustrate disinvestment and resource allocation decision-making processes in health care: A systematic review. International journal of technology assessment in health care. 2013;29(2):174-84. doi:10.1017/s0266462313000068.

2. Marks L, Weatherly H, Mason A. Prioritizing investment in public health and health equity: what can commissioners do? Public health. 2013;127(5):410-8. doi:10.1016/j.puhe.2013.01.027.

3. Smith N, Mitton C, Peacock S, Cornelissen E, MacLeod S. Identifying research priorities for health care priority setting: a collaborative effort between managers and researchers. BMC health services research. 2009;9:165. doi:10.1186/1472-6963-9-165.

4. Harris C, Allen K, Waller C, Voutier C, Brooke V. Health technology disinvestment: tests, drugs and clinical practice. Report on a national disinvestment workshop. Part 1: Report. Centre for Clinical Effectiveness 2009. Available from: http://arrow.monash.edu.au/hdl/1959.1/1218926. Accessed: October 2016

5. Harris C, Allen K, Waller C, Green S, King R, Ramsey W et al. Sustainability in Health care by Allocating Resources Effectively (SHARE) 5: Developing a model for evidence-driven resource allocation in the local healthcare setting BMC health services research. 2017;(Details TBA).

6. Mitton C, Dionne F, Donaldson C. Managing healthcare budgets in times of austerity: the role of program budgeting and marginal analysis. Applied health economics and health policy. 2014;12(2):95-102. doi:10.1007/s40258-013-0074-5.

7. Robinson S, Glasby J, Allen K. 'It ain't what you do it's the way that you do it': lessons for health care from decommissioning of older people's services. Health & social care in the community. 2013;21(6):614-22. doi:10.1111/hsc.12046.

8. Robinson S, Williams I, Dickinson H, Freeman T, Rumbold B. Priority-setting and rationing in healthcare: evidence from the English experience. Social science & medicine. 2012;75(12):2386-93. doi:10.1016/j.socscimed.2012.09.014.

9. Schmidt DE. The development of a disinvestment framework to guide resource allocation decisions in health service delivery organizations. The University of British Columbia 2010. Available from: https://open.library.ubc.ca/cIRcle/collections/ubctheses/24/items/1.0073252. Accessed: October 2016

10. Eddama O, Coast J. A systematic review of the use of economic evaluation in local decision-making. Health policy (Amsterdam, Netherlands). 2008;86(2-3):129-41. doi:10.1016/j.healthpol.2007.11.010.

11. Department of Human Services. Future directions for health technology uptake, diffusion and disinvestment in Victorian public health services. Department of Human Services, Victoria, Australia. 2007.

12. Mayer J, Nachtnebel A. Disinvesting from ineffective technologies: Lessons learned from current programs. International journal of technology assessment in health care. 2015:1-8. doi:10.1017/s0266462315000641.

13. Transplantation Society of Australia and New Zealand. Organ transplantation from deceased donors: Consensus statement on eligibility criteria and allocation protocols. 2014. Available from: http://www.donatelife.gov.au/sites/default/files/files/Concensus_Statement_v3.pdf. Accessed: September 2015

14. NHS Commissioning Board. Commissioning Policy: Ethical framework for priority setting and resource allocation 2013. Available from: http://www.england.nhs.uk/wp-content/uploads/2013/04/cp-01.pdf. Accessed: October 2016

15. Indiana University Center for Bioethics. Ethics Guide for Health Care Practitioners. Working Under Conditions of an Influenza Pandemic. 2009. Available from: https://scholarworks.iupui.edu/bitstream/handle/1805/2011/EthicsGuide-Pandemic_Pocket.pdf?sequence=1&isAllowed=y. Accessed: September 2015

16. Lindstrom H, Waldau S. Ethically acceptable prioritisation of childless couples and treatment rationing: "accountability for reasonableness". European journal of obstetrics, gynecology, and reproductive biology. 2008;139(2):176-86. doi:10.1016/j.ejogrb.2008.02.018.

17. World Health Organisation. Guiding principles on human cell, tissue and organ transplantation. 2008. Available from: http://www.who.int/transplantation/Guiding_PrinciplesTransplantation_WHA63.22en.pdf. Accessed: September 2015

18. World Health Organisation. Guidance on ethics and equitable access to HIV treatment and care. 2004. Available from: http://www.who.int/hiv/pub/advocacy/en/guidanceethics_en.pdf?ua=1. Accessed: September 2015

19. National Health and Medical Research Council. Ethical considerations relating to health care resource allocation decisions. Commonwealth of Australia 1993. Available from: https://www.nhmrc.gov.au/_files_nhmrc/publications/attachments/e24.pdf. Accessed: October 2015

20. Rawlins MD. Evidence, values, and decision making. International journal of technology assessment in health care. 2014:1-6. doi:10.1017/s0266462314000154.

21. Culyer A. Efficiency, equity and equality in health and health care. Centre for Health Economics, University of York, UK, 2015.

22. Peacock SJ, Mitton C, Ruta D, Donaldson C, Bate A, Hedden L. Priority setting in healthcare: towards guidelines for the program budgeting and marginal analysis framework. Expert review of pharmacoeconomics & outcomes research. 2010;10(5):539-52. doi:10.1586/erp.10.66.

23. Hauck K, Smith P, Goddard M. The Economics of Priority Setting for Health Care: A Literature Review. Health, Nutrition and Population Discussion Paper World Bank 2004. Available from: http://siteresources.worldbank.org/HEALTHNUTRITIONANDPOPULATION/Resources/281627-1095698140167/Chapter3Final.pdf. Accessed: October 2016

24. Daniels N, Sabin J. Limits to health care: fair procedures, democratic deliberation, and the legitimacy problem for insurers. Philosophy & public affairs. 1997;26(4):303-50.

25. HealthPACT. Disinvestment in Australia and New Zealand. Health Policy Advisory Committee on Technology 2013. Available from: http://www.health.qld.gov.au/healthpact/docs/papers/workshop/disinvestment-report.pdf. Accessed: October 2016

26. Guindo LA, Wagner M, Baltussen R, Rindress D, van Til J, Kind P et al. From efficacy to equity: Literature review of decision criteria for resource allocation and healthcare decisionmaking. Cost effectiveness and resource allocation : C/E. 2012;10(1):9. doi:10.1186/1478-7547-10-9.

27. Cornelissen E, Mitton C, Davidson A, Reid RC, Hole R, Visockas AM et al. Changing priority setting practice: The role of implementation in practice change. Health policy (Amsterdam, Netherlands). 2014. doi:10.1016/j.healthpol.2014.04.010.

28. Evans BA, Snooks H, Howson H, Davies M. How hard can it be to include research evidence and evaluation in local health policy implementation? Results from a mixed methods study. Implementation science : IS. 2013;8:17. doi:10.1186/1748-5908-8-17.

29. Rumbold B, Alakeson V, Smith P. Rationing health care. Quest for NHS Efficiency Series 2012. Available from: http://www.nuffieldtrust.org.uk/sites/files/nuffield/publication/rationing_health_care_240212_0.pdf. Accessed: October 2016

30. MacKean G, Noseworthy T, Elshaug AG, Leggett L, Littlejohns P, Berezanski J et al. Health technology reassessment: The art of the possible. International journal of technology assessment in health care. 2013;29(04):418-23. doi:doi:10.1017/S0266462313000494.

31. Robert G, Harlock J, Williams I. Disentangling rhetoric and reality: an international Delphi study of factors and processes that facilitate the successful implementation of decisions to decommission healthcare services. Implementation science : IS. 2014;9(1):123. doi:10.1186/s13012-014-0123-y.

32. Henshall C, Schuller T, Mardhani-Bayne L. Using health technology assessment to support optimal use of technologies in current practice: the challenge of "disinvestment". International journal of technology assessment in health care. 2012;28(3):203-10. doi:10.1017/s0266462312000372.

33. Harris C, Allen K, King R, Ramsey W, Kelly C, Thiagarajan M. Sustainability in Health care by Allocating Resources Effectively (SHARE) 2: Identifying opportunities for disinvestment in a local healthcare setting BMC health services research. 2017;(Details TBA).

34. Fronsdal KB, Facey K, Klemp M, Norderhaug IN, Morland B, Rottingen JA. Health technology assessment to optimize health technology utilization: using implementation initiatives and monitoring processes. International journal of technology assessment in health care. 2010;26(3):309-16. doi:10.1017/s0266462310000309.

35. Armstrong R, Waters E, Dobbins M, Anderson L, Moore L, Petticrew M et al. Knowledge translation strategies to improve the use of evidence in public health decision making in local government: intervention design and implementation plan. Implementation science : IS. 2013;8(1):121. doi:10.1186/1748-5908-8-121.

36. Harris C, Allen K, Waller C, Brooke V. Sustainability in Health care by Allocating Resources Effectively (SHARE) 3: Examining how resource allocation decisions are made, implemented and evaluated in a local healthcare setting BMC health services research. 2017;(Details TBA).

37. Wiltsey Stirman S, Kimberly J, Cook N, Calloway A, Castro F, Charns M. The sustainability of new programs and innovations: a review of the empirical literature and recommendations for future research. Implementation science : IS. 2012;7:17. doi:10.1186/1748-5908-7-17.

38. Organisation for Economic Co-operation and Development. The OECD Health Project: Health Technologies and Decision Making. OECD Publishing 2005. Available from: http://www.keepeek.com/Digital-Asset-Management/oecd/science-and-technology/health-technologies-and-decision-making_9789264016224-en#page1. Accessed: September 2015

39. Hughes E, McKenny K. Decommissioning and Disinvestment Toolkit 2013-2014 Castle Point and Rochford Clinical Commissioning Group 2013. Available from: http://castlepointandrochfordccg.nhs.uk/about-us/key-documents/policies/corporate-policies/299-decommissioning-and-disinvestment-strategy/file. Accessed: October 2016

40. Harris C, Garrubba M, Allen K, King R, Kelly C, Thiagarajan M et al. Development, implementation and evaluation of an evidence-based program for introduction of new health technologies and clinical practices in a local healthcare setting. BMC health services research. 2015;15(1):575. doi:10.1186/s12913-015-1178-4.

41. NHS Confederation. Two sides of the same coin. Balancing quality and finance to deliver greater value. 2014. Available from: http://www.nhsconfed.org/~/media/Confederation/Files/Publications/Documents/two_sides_same_coin150413.pdf. Accessed: October 2016

42. Sibbald SL, Singer PA, Upshur R, Martin DK. Priority setting: what constitutes success? A conceptual framework for successful priority setting. BMC health services research. 2009;9:43. doi:10.1186/1472-6963-9-43.

43. Stafinski T, Menon D, McCabe C, Philippon DJ. To fund or not to fund: development of a decision-making framework for the coverage of new health technologies. Pharmacoeconomics. 2011;29(9):771-80. doi:10.2165/11539840-000000000-00000.

44. Smith N, Mitton C, Bryan S, Davidson A, Urquhart B, Gibson JL et al. Decision maker perceptions of resource allocation processes in Canadian health care organizations: a national survey. BMC health services research. 2013;13:247. doi:10.1186/1472-6963-13-247.

45. Robinson S, Dickinson H, Williams I, Freeman T, Rumbold B, Spence K. Setting priorities in health: A study of English primary care trusts: Health Services Management Centre, University of Birmingham and the Nuffield Trust, 2011.

46. Daniels N, Sabin J. Accountability for reasonableness: an update. BMJ. 2008;337. doi:10.1136/bmj.a1850.

47. Barasa EW, Molyneux S, English M, Cleary S. Setting Healthcare Priorities at the Macro and Meso Levels: A Framework for Evaluation. International journal of health policy and management. 2015;4(11):719-32. doi:10.15171/ijhpm.2015.167.

48. Durand MA, Carpenter L, Dolan H, Bravo P, Mann M, Bunn F et al. Do interventions designed to support shared decision-making reduce health inequalities? A systematic review and meta-analysis. PLoS One. 2014;9(4):e94670. doi:10.1371/journal.pone.0094670.

49. Ryan R, Santesso N, Lowe D, Hill S, Grimshaw J, Prictor M et al. Interventions to improve safe and effective medicines use by consumers: an overview of systematic reviews. Cochrane database of systematic reviews (Online). 2014;4:CD007768. doi:10.1002/14651858.CD007768.pub3.

50. Sarrami-Foroushani P, Travaglia J, Debono D, Braithwaite J. Key concepts in consumer and community engagement: a scoping meta-review. BMC health services research. 2014;14:250. doi:10.1186/1472-6963-14-250.

51. Oxman AD, Lewin S, Lavis JN, Fretheim A. SUPPORT Tools for evidence-informed health Policymaking (STP) 15: Engaging the public in evidence-informed policymaking. Health research policy and systems / BioMed Central. 2009;7 Suppl 1:S15. doi:10.1186/1478-4505-7-s1-s15.

52. Boivin A, Lehoux P, Lacombe R, Burgers J, Grol R. Involving patients in setting priorities for healthcare improvement: a cluster randomized trial. Implementation science : IS. 2014;9:24. doi:10.1186/1748-5908-9-24.

53. O'Mara-Eves A, Brunton G, McDaid D, Oliver S, Kavanagh J, Jamal F et al. Community engagement to reduce inequalities in health: a systematic review, meta-analysis and economic analysis. Public Health Res. 2013;1(4).

54. Nilsen ES, Myrhaug HT, Johansen M, Oliver S, Oxman AD. Methods of consumer involvement in developing healthcare policy and research, clinical practice guidelines and patient information material. Cochrane database of systematic reviews (Online). 2006(3):CD004563. doi:10.1002/14651858.CD004563.pub2.

55. Abelson J, Gauvin FP. Engaging Citizens: One Route to Health Care Accountability. Ottawa, 2004.

56. Gagnon MP, Desmartis M, Gagnon J, St-Pierre M, Rhainds M, Coulombe M et al. Framework for user involvement in health technology assessment at the local level: Views of health managers, user representatives, and clinicians. International journal of technology assessment in health care. 2015;31(1-2):68-77. doi:10.1017/s0266462315000070.

57. Harris C, Ko H, Waller C, Sloss P, Williams P. Sustainability in Health care by Allocating Resources Effectively (SHARE) 4: Exploring opportunities and methods for consumer engagement in resource allocation in a local healthcare setting BMC health services research. 2017;(Details TBA).

58. Abelson J, Giacomini M, Lehoux P, Gauvin FP. Bringing 'the public' into health technology assessment and coverage policy decisions: from principles to practice. Health policy (Amsterdam, Netherlands). 2007;82(1):37-50. doi:10.1016/j.healthpol.2006.07.009.

59. Wolfson D, Santa J, Slass L. Engaging physicians and consumers in conversations about treatment overuse and waste: a short history of the choosing wisely campaign. Academic medicine : journal of the Association of American Medical Colleges. 2014;89(7):990-5. doi:10.1097/acm.0000000000000270.

60. Hodgetts K, Hiller JE, Street JM, Carter D, Braunack-Mayer AJ, Watt AM et al. Disinvestment policy and the public funding of assisted reproductive technologies: outcomes of deliberative engagements with three key stakeholder groups. BMC health services research. 2014;14:204. doi:10.1186/1472-6963-14-204.

61. Watt AM, Willis CD, Hodgetts K, Elshaug AG, Hiller JE. Engaging clinicians in evidence-based disinvestment: role and perceptions of evidence. International journal of technology assessment in health care. 2012;28(03):211-9. doi:doi:10.1017/S0266462312000402.

62. Watt AM, Hiller JE, Braunack-Mayer AJ, Moss JR, Buchan H, Wale J et al. The ASTUTE Health study protocol: deliberative stakeholder engagements to inform implementation approaches to healthcare disinvestment. Implementation science : IS. 2012;7:101. doi:10.1186/1748-5908-7-101.

63. Hollingworth W, Rooshenas L, Busby J, Hine CE, Badrinath P, Whiting PF et al. Using clinical practice variations as a method for commissioners and clinicians to identify and prioritise opportunities for disinvestment in health care: a cross-sectional study, systematic reviews and qualitative study. Southampton UK: Queen's Printer and Controller of HMSO 2015; 2015.

64. Haas M, Hall J, Viney R, Gallego G. Breaking up is hard to do: why disinvestment in medical technology is harder than investment. Australian health review : a publication of the Australian Hospital Association. 2012;36(2):148-52. doi:10.1071/AH11032.

65. Gerdvilaite J, Nachtnebel A. Disinvestment: overview of disinvestment experiences and challenges in selected countries. HTA-Projektbericht., vol Nr. 57. Ludwig Boltzmann Institut für Health Technology Assessment; 2011.

66. Ibargoyen-Roteta N, Gutiérrez-Ibarluzea I, Asua J. Report on the development of the GuNFT Guideline. Guideline for Not Funding existing health Technologies in health care systems. Quality Plan for the NHS of the MHSP. Basque Office for Health Technology Assessment (Osteba). 2009;Reports of Health Technology Assessment. Osteba Nº 2007/11.

67. Iglesias CP, Drummond MF, Rovira J. Health-care decision-making processes in Latin America: problems and prospects for the use of economic evaluation. International journal of technology assessment in health care. 2005;21(1):1-14.

68. Massatti RR, Sweeney HA, Panzano PC, Roth D. The de-adoption of innovative mental health practices (IMHP): why organizations choose not to sustain an IMHP. Administration and policy in mental health. 2008;35(1-2):50-65. doi:10.1007/s10488-007-0141-z.

69. Elshaug AG, Hiller JE, Tunis SR, Moss JR. Challenges in Australian policy processes for disinvestment from existing, ineffective health care practices. Australia and New Zealand health policy. 2007;4:23. doi:10.1186/1743-8462-4-23.

70. Williams I, Bryan S, McIver S. The use of economic evaluations in NHS decision making: A review and empirical investigation: Health Economics Facility, University of Birmingham, Health Services Management Centre, University of Birmingham, 2006.

71. Harris C, Allen K, Waller C, Dyer T, Brooke V, Garrubba M et al. Sustainability in Health care by Allocating Resources Effectively (SHARE) 7: Supporting staff in evidence-based decision-making, implementation and evaluation in a local healthcare setting BMC health services research. 2017;(Details TBA).

72. Ellen ME, Leon G, Bouchard G, Ouimet M, Grimshaw JM, Lavis JN. Barriers, facilitators and views about next steps to implementing supports for evidence-informed decision-making in health systems: a qualitative study. Implementation science : IS. 2014;9(1):179. doi:10.1186/s13012-014-0179-8.

73. Mortimer D. Reorienting programme budgeting and marginal analysis (PBMA) towards disinvestment. BMC health services research. 2010;10:288. doi:10.1186/1472-6963-10-288.

74. Decamp M, Farber NJ, Torke AM, George M, Berger Z, Keirns CC et al. Ethical Challenges for Accountable Care Organizations: A Structured Review. Journal of general internal medicine. 2014. doi:10.1007/s11606-014-2833-x.

75. Rubenfeld GD. Cost-effective critical care: cost containment and rationing. Seminars in respiratory and critical care medicine. 2012;33(4):413-20. doi:10.1055/s-0032-1322411.

76. Elshaug AG, McWilliams J, Landon BE. The value of low-value lists. JAMA. 2013;309(8):775-6. doi:10.1001/jama.2013.828.

77. Tromp N, Baltussen R. Mapping of multiple criteria for priority setting of health interventions: an aid for decision makers. BMC health services research. 2012;12:454. doi:10.1186/1472-6963-12-454.

78. Singer PA, Martin DK, Giacomini M, Purdy L. Priority setting for new technologies in medicine: qualitative case study. BMJ. 2000;321(7272):1316-8.

79. Panteli D, Kreis J, Busse R. Considering equity in health technology assessment: An exploratory analysis of agency practices. International journal of technology assessment in health care. 2015;31(5):314-23. doi:10.1017/s0266462315000549.

80. Levesque JF, Harris MF, Russell G. Patient-centred access to health care: conceptualising access at the interface of health systems and populations. International journal for equity in health. 2013;12:18. doi:10.1186/1475-9276-12-18.

81. Rooshenas L, Owen-Smith A, Hollingworth W, Badrinath P, Beynon C, Donovan JL. "I won't call it rationing...": an ethnographic study of healthcare disinvestment in theory and practice. Social science & medicine. 2015;128:273-81. doi:10.1016/j.socscimed.2015.01.020.

82. Healthcare Improvement Scotland. What approaches have been taken and efforts made to ensure public involvement in decision making relating to potential disinvestment in healthcare interventions and technologies? Technologies scoping report 16. 2013. Available from: file:///C:/Users/claireha/Downloads/Disinvestment%20(2).pdf. Accessed: June 2015

83. Garner S, Littlejohns P. Disinvestment from low value clinical interventions: NICEly done? BMJ. 2011;343(jul27 2):d4519-d. doi:10.1136/bmj.d4519.

84. Audit Commission. Reducing spending on low clinical value treatments. Health Briefing. London, 2011

85. Leggett L, MacKean G, Noseworthy T, Sutherland L, Clement F. Current status of health technology reassessment of non-drug technologies: survey and key informant interviews. Health Research Policy and Systems 2012. doi:10.1186/1478-4505-10-38.

86. Elshaug A, Moss J, Littlejohns P, Karnon J, Merlin T, Hiller J. Identifying existing health care services that do not provide value for money. Medical Journal of Australia. 2009;190(5):269-73.

87. Poulin P, Austen L, Scott CM, Poulin M, Gall N, Seidel J et al. Introduction of new technologies and decision making processes: a framework to adapt a Local Health Technology Decision Support Program for other local settings. Medical devices (Auckland, NZ). 2013;6:185-93. doi:10.2147/mder.s51384.

88. Garcia-Armesto S, Campillo-Artero C, Bernal-Delgado E. Disinvestment in the age of cost-cutting sound and fury. Tools for the Spanish National Health System. Health policy (Amsterdam, Netherlands). 2013;110(2-3):180-5. doi:10.1016/j.healthpol.2013.01.007.

89. Leggett L, Noseworthy TW, Zarrabi M, Lorenzetti D, Sutherland LR, Clement FM. Health technology reassessment of non-drug technologies: current practices. International journal of technology assessment in health care. 2012;28(3):220-7. doi:10.1017/S0266462312000438.

90. Noseworthy T, Clement F. Health technology reassessment: Scope, methodology, & language. International journal of technology assessment in health care. 2012;28(03):201-2. doi:doi:10.1017/S0266462312000359.

91. Mitchell MD, Williams K, Brennan PJ, Umscheid CA. Integrating local data into hospital-based healthcare technology assessment: two case studies. International journal of technology assessment in health care. 2010;26(3):294-300. doi:10.1017/s0266462310000334.

92. Ruano-Ravina A, Velasco-Gonzalez M, Varela-Lema L, Cerda-Mota T, Ibargoyen-Roteta N, Gutierrez-Ibarluzea I et al. Identification, prioritisation and assessment of obsolete health technologies. A methodolgical guideline. Quality Plan for the National Health System. 2009;Galician Health Technology Assessment Agency(HTA Reports: avalia-t No. 2007/01).

93. Segal L, Mortimer D. A population-based model for priority setting across the care continuum and across modalities. Cost Effectiveness & Resource Allocation. 2006;4:6.

94. Bryan S, Mitton C, Donaldson C. Breaking the addiction to technology adoption. Health Economics. 2014;23(4):379-83. doi:10.1002/hec.3034.

95. Donaldson C, Bate A, Mitton C, Dionne F, Ruta D. Rational disinvestment. QJM. 2010;103(10):801-7. doi:10.1093/qjmed/hcq086.

96. Daniels T, Williams I, Robinson S, Spence K. Tackling disinvestment in health care services. The views of resource allocators in the English NHS. Journal of health organization and management. 2013;27(6):762-80.

97. Ibargoyen-Roteta N, Gutierrez-Ibarluzea I, Asua J. Guiding the process of health technology disinvestment. Health policy (Amsterdam, Netherlands). 2010;98(2-3):218-26. doi:10.1016/j.healthpol.2010.06.018.

98. Rubinstein A, Belizan M, Discacciati V. Are economic evaluations and health technology assessments increasingly demanded in times of rationing health services? The case of the Argentine financial crisis. International journal of technology assessment in health care. 2007;23(2):169-76. doi:10.1017/s0266462307070274.

99. National Health Committee NZ. Business Plan 2013/14-2015/16. Wellington, 2013

100. Blumenthal-Barby JS. “Choosing Wisely” to Reduce Low-Value Care: A Conceptual and Ethical Analysis. Journal of Medicine and Philosophy. 2013;38(5):559-80. doi:10.1093/jmp/jht042.

101. Liverani M, Hawkins B, Parkhurst JO. Political and institutional influences on the use of evidence in public health policy. A systematic review. PLoS One. 2013;8(10):e77404. doi:10.1371/journal.pone.0077404.

102. Jewell CJ, Bero LA. "Developing good taste in evidence": facilitators of and hindrances to evidence-informed health policymaking in state government. The Milbank quarterly. 2008;86(2):177-208. doi:10.1111/j.1468-0009.2008.00519.x.

103. Garner S, Docherty M, Somner J, Sharma T, Choudhury M, Clarke M et al. Reducing ineffective practice: challenges in identifying low-value health care using Cochrane systematic reviews. Journal of health services research & policy. 2013;18(1):6-12. doi:10.1258/jhsrp.2012.012044.

104. Vogel JP, Oxman AD, Glenton C, Rosenbaum S, Lewin S, Gulmezoglu AM et al. Policymakers' and other stakeholders' perceptions of key considerations for health system decisions and the presentation of evidence to inform those considerations: an international survey. Health research policy and systems / BioMed Central. 2013;11:19. doi:10.1186/1478-4505-11-19.

105. Bastian H, Scheibler F, Knelangen M, Zschorlich B, Nasser M, Waltering A. Choosing health technology assessment and systematic review topics: the development of priority-setting criteria for patients' and consumers' interests. International journal of technology assessment in health care. 2011;27(4):348-56. doi:10.1017/s0266462311000547.

106. Larmour I, Pignataro S, Barned KL, Mantas S, Korman MG. A therapeutic equivalence program: evidence-based promotion of more efficient use of medicines. The Medical journal of Australia. 2011;194(12):631-4.

107. IAP2. Foundations of Public Participation. International Association for Public Participation. Available from: http://www.iap2.org.au/documents/item/83. Accessed: May 2015

108. National Collaborating Centre for Methods and Tools. Engaging citizens for decision making. McMaster University, Hamilton. 2011. http://www.nccmt.ca/registry/view/eng/86.html.

109. Scottish Health Council. The Participation Toolkit. Supporting Patient Focus and Public Involvement in NHS Scotland. 2014. Available from: http://shsc.nhs.uk/wp-content/uploads/2014/05/ParticipationToolkit.pdf. Accessed: June 2015

110. Health Consumers Queensland. Developing a consumer and community engagement strategy: a toolkit for Hospital and Health Services. Queensland Health 2012. Available from: http://www.health.qld.gov.au/hcq/publications/hcq-toolkit.pdf. Accessed: June 2015

111. Austin Health. Engaging with our Consumers. Austin Health 2013. Available from: http://www.austin.org.au/Assets/Files/CE%20Plan%20FINAL.pdf. Accessed: June 2015

112. CanNET Victoria. Consumer Participation Toolkit. Victorian Integrated Cancer Services Consumer Participation Network 2012. Available from: http://www.gha.net.au/Uploadlibrary/410266204VICConsumerParticipationToolkitFINAL.pdf. Accessed: June 2015

113. Cowan K, Oliver S. The James Lind Alliance Guidebook. Southhampton: James Lind Alliance; 2013.

114. World Economic Forum. Multistakeholder Collaboration for Healthy Living. Toolkit for Joint Action. 2013. Available from: http://www3.weforum.org/docs/WEF_HE_HealthyLiving_Toolkit_2013.pdf. Accessed:

115. MacQueen K, Harlan S, Slevin K, Hannah S, Bass E, Moffett J. Stakeholder Engagement Toolkit. U.S. Agency for International Development and FHI 360 2012. Available from: http://www.fhi360.org/sites/default/files/media/documents/Stakeholder%20EngagementToolkit%20for%20HIV%20Prevention%20Trials.pdf. Accessed: September 2015

116. Jeyanathan T, Dhalla I, Culyer T, Levinson W, Laupacis A, Martin D et al. Recommendations for Establishing a Citizens' Council to Guide Drug Policy in Ontario. Toronto: Institute for Clinical Evaluative Sciences 2006

117. Boivin A, Lehoux P, Burgers J, Grol R. What Are the Key Ingredients for Effective Public Involvement in Health Care Improvement and Policy Decisions? A Randomized Trial Process Evaluation. Milbank Quarterly. 2014;92(2):319-50. doi:10.1111/1468-0009.12060.

118. Chisholm A, Briggs K, Askham J. Not NICE. Can PCTs engage patients and the public in commissioning new health technologies? Oxford: Picker Institute Europe, 2009

119. Picker Institute. Patient and public engagement – the early impact of World Class Commissioning. A Survey of Primary Care Trusts Oxford: Picker Institute Europe, 2009

120. Solomons NM, Spross JA. Evidence-based practice barriers and facilitators from a continuous quality improvement perspective: an integrative review. Journal of nursing management. 2011;19(1):109-20. doi:10.1111/j.1365-2834.2010.01144.x.

121. Harris C, Garrubba M, Melder A, Voutier C, Waller C, King R et al. Sustainability in Health care by Allocating Resources Effectively (SHARE) 8: Developing, implementing and evaluating an Evidence Dissemination Service in a local healthcare setting. BMC health services research. 2017;(Details TBA).

122. Harris C, Allen K, Brooke V, Dyer T, Waller C, King R et al. Sustainability in Health care by Allocating Resources Effectively (SHARE) 6: Investigating methods to identify, prioritise, implement and evaluate disinvestment projects in a local healthcare setting. BMC health services research. 2017;(Details TBA).
